# Metabolic Profiling Comparison of Human Pancreatic Ductal Epithelial Cells and Three Pancreatic Cancer Cell Lines using NMR Based Metabonomics

**DOI:** 10.4172/2155-9929.S3-002

**Published:** 2012-03-12

**Authors:** Miki Watanabe, Sulaiman Sheriff, Kenneth B. Lewis, Junho Cho, Stuart L. Tinch, Ambikaipakan Balasubramaniam, Michael A. Kennedy

**Affiliations:** 1 Department of Chemistry and Biochemistry, Miami University, Oxford, Ohio, USA; 2 Department of surgery, University of Cincinnati Medical Center, Cincinnati, Ohio, USA; 3 Shriners Hospital for Children, Cincinnati, OH 45229, USA; 4 Cincinnati Veterans Affairs Medical Center, Cincinnati, OH 45220, USA

**Keywords:** Pancreatic cancer, Miapaca-2, Panc-1, AsPC-1, H6C7, NMR, Metabonomics, Metabolic profiling, PCA

## Abstract

Metabolic profiles of hydrophilic and lipophilic cell extracts from three cancer cell lines, Miapaca-2, Panc-1 and AsPC-1, and a non-cancerous pancreatic ductal epithelial cell line, H6C7, were examined by proton nuclear magnetic resonance spectroscopy. Over twenty five hydrophilic metabolites were identified by principal component and statistical significance analyses as distinguishing the four cell types. Fifteen metabolites were identified with significantly altered concentrations in all cancer cells, e.g. absence of phosphatidylgrycerol and phosphatidylcholine, and increased phosphatidylethanolamine and cholesterols. Altered concentrations of metabolites involved in glycerophospholipid metabolism, lipopolysaccharide and fatty acid biosynthesis indicated differences in cellular membrane composition between non-cancerous and cancer cells. In addition to cancer specific metabolites, several metabolite changes were unique to each cancer cell line. Increased N-acetyl groups in AsPC-1, octanoic acids in Panc-1, and UDP species in Miapaca-2 indicated differences in cellular membrane composition between the cancer cell lines. Induced glutamine metabolism and protein synthesis in cancer cells were indicated by absence of glutamine other metabolites such as acetate, lactate, serine, branched amino acids, and succinate. Knowledge of the specifically altered metabolic pathways identified in these pancreatic cancer cell lines may be useful for identifying new therapeutic targets and studying the effects of potential new therapeutic drugs.

## Introduction

Pancreatic cancer is one of the most lethal human cancers. According to the cancer statistics from 2010, more than 40 thousand patients are expected to be diagnosed in coming year [1]. Pancreatic cancer has the lowest five-year survival rate among all types of cancers. One of the reasons for such high mortality is the lack of early detection and diagnostic methods [2]. Most diagnoses occur after the cancer has progressed to an advanced stage when a surgical cure is not feasible. Biopsies, tomography scans, endoscopic ultrasound and endoscopic retrograde cholangiopancreatography are some of the current detection and diagnostic methods for pancreatic cancer [2,3]. However, these methods are highly invasive and not suitable for general population screening. Development of an early detection method for pancreatic cancer is essential in order to improve the likelihood that diagnosis will occur before the cancer is at a stage that is too advanced to treat.

Metabonomics is a non-invasive method used to identify biomarkers in biofluids such as urine, serum, plasma, stool, saliva and blood. Nuclear magnetic resonance (NMR) spectroscopy, gas chromatography mass spectrometry (GC-MS), liquid chromatography mass spectrometry (LC-MS), and capillary electrophoresis mass spectrometry are a few of the techniques used for metabolic profiling in both research and clinical applications [4]. For example, GC-MS has been used to differentiate between invasive ovarian carcinomas and borderline tumors in flash frozen human tumor samples (reviewed in Griffin et al.) [5]. Sarcosine was suggested as a potential biomarker for prostate cancer progression by LC-MS based metabonomics analysis [6]. By using NMR spectroscopy, several metabolites such as lactate in blood plasma and creatinine and phenylacetylglycine in urine were identified as potential biomarkers for lung cancer [7]. In pancreatic cancer research, a number of studies have recently reported the potential identification of early biomarkers in biofluids using metabolic profiling [8–11]. Despite these encouraging studies, individual variability remains a challenge for the discovery and validation of disease biomarkers in human samples.

Metabonomics can be used not only for biofluids, but also for cell cultures and tissues. As is the case in most cancer research, the majority of pancreatic cancer studies are based on *in vitro* examinations using various cell lines. Cancer cell lines are widely used to study potential therapeutic drugs, to identify proteins with altered expression levels, gene mutations, and inactivation or activation of metabolic pathways [12–14]. Several metabonomics studies have shown differences in metabolic profiles among different cancer cell lines [15] and the metabolic processes that occur during apoptosis and cell cycle phases [16,17]. In addition, monitoring metabolic profiles can also be useful to study the effect of various stimuli such as drug treatments [18,19].

Even though findings from cancer cell line studies have been presented as generally characteristic of the disease, heterogeneity in both the pathology of patients and between pancreatic cancer cell lines must be anticipated. Sipos et al. have categorized the cellular structure, population doubling time, and functional marker expressions of twelve pancreatic ductal adenocarcinoma cell lines [20]. In addition, Monti et al. [21] further showed differences in expression of immunorelevant molecules, secretion of immunomodulatory cytokines and susceptibility to apoptosis and chemotherapeutic agents [21].

Three human pancreatic cancer cell lines, Miapaca-2, Panc-1 and AsPC-1, were used in this study and table 1 provides a summary of the characteristics of the cell lines. According to these comparative studies, the Miapaca-2 cell line originated from a patient with a primary adenocarcinoma tumor and has epithelial cell like morphology. These cells are poorly differentiated and have a large abundant cytoplasm. It has been shown that the doubling time of Miapaca-2 is about 40 hours. Panc-1 cells are often used as an *in vitro* model of non-endocrine pancreatic cancer for tumorigenicity studies. These cells were isolated from a primary tumor in the pancreatic duct of an epithelioid carcinoma patient. Panc-1 also shows epithelial cell like morphology and is poorly differentiated. The doubling time of Panc-1 is around 56 hours. AsPC-1 cells originated from pancreas ascites from an adenocarcinoma patient. These cells also have the epithelial cell like morphology but are moderately to highly differentiated with the doubling time of about 58 hours. In this study, the metabolic profiles of these three pancreatic cancer cell lines were examined by NMR spectroscopy and compared with immortalized human pancreatic ductal epithelial cells.

Because the majority (∼90%) of human pancreatic cancers belong to an adenocarcinoma of ductal epithelial origin, a non-cancerous immortalized human pancreatic ductal epithelial (HPDE) cell line HPDE6-E6E76c7 (H6C7) [22] was used as a control in this study. NMR spectra of hydrophilic and lipophilic extracts from three human cancer cell lines, Miapaca-2, Panc-1, and AsPC-1 were compared to the control to identify unique metabolic profiling characteristics of each cell line. This study provides not only potential biomarkers for pancreatic cancer, but also provides insight into the metabolic profiles of cell lines often used for *in vivo* therapeutic studies. These metabonomic profiles can be further used to study the effect of the potential therapeutic drugs.

## Materials and Methods

### Pancreatic cancer cell line cultures

Human pancreatic adenocarcinoma cells, Panc-1, Miapaca-2 and AsPC1, were obtained from American Type Culture Collection (Manassas, VA). The immortalized HDPE cell line HPDE6-E6E76c7 (H6C7) was obtained from Dr. Tsao (Ontario cancer institute/Princess Margaret hospital, University Health Network). Miapaca-1 and Panc-1 cells were grown in high glucose Dulbecco’s Modified Eagle Medium (Hyclone) supplemented with 10% fetal bovine serum (FBS), penicillin and streptomycin. Cells were maintained in a 5% CO_2_, humidified atmosphere at 37°C. AsPC1 cells were grown in Gibco® RPMI medium (Invitrogen) supplemented with 10% FBS, penicillin and streptomycin. H6C7 cells were maintained in keratinocyte serum free media supplemented with epidermal growth factor and bovine pituitary extract (Invitrogen). Cells were maintained in 5% CO_2_, humidified atmosphere at 37°C. Confluent cells were trypsinized and seeded into 100 mm dishes and maintained. When cells were grown to 75–85% confluence in 10cm culture dishes, the media was aspirated and cells were trypsinized using trypsin-EDTA solution (Invitrogen). Fresh growth media containing 10% FBS was added to the trypsinized cells (10:1 ratio) and centrifuged for 5 minutes at 1,500 rpm at 4°C. The media was aspirated and the cell pellet was washed with 10 ml of cold phosphate buffered saline, pH 7.4, and centrifuged again as above. This step was repeated two more times and the cell pellets were stored at −80°C until removal for cell extraction.

### Cell extraction

Methanol-chloroform-water extraction was performed as previously described [23]. Briefly, cell pellets were resuspended in 500 µL of ice-cold 2:1 (v/v) methanol:chloroform solution and then transferred into a 1.5 ml Eppendorf tube. After vortexing, the tubes were incubated on a mixer for 10 min at 4°C. Then, 250 µl of ice-cold H_2_O and 250 µl of chloroform were added to the cells and mixed using a vortex mixer. The tubes were sonicated in a water bath for 10 min and centrifuged for 10 min at 17949xg. The top layer, i.e. the hydrophilic extract, and bottom layer, i.e. the lipophilic extract, were transferred into new Eppendorf tubes. The samples were dried in a SpeedVac centrifuge for 2–3 h and stored at −20°C until further preparation for NMR analysis.

### NMR spectroscopy

Hydrophilic cell extracts were resuspended in 200 µL of buffer (150 mM potassium phosphate at pH 7.4, 1 mM NaN_3_, 0.01 % trimethylsilylpropionate (TSP) in 100% D_2_O (the required quantity of buffer for each sample was originally prepared in H_2_O, lyophilized, the reconstituted in 100% D_2_O) and lipophilic cell extracts were resuspended in 200 µL deuterated chloroform. All NMR spectra were recorded on a Bruker Avance™ III spectrometer operating at 850.1 MHz. All experiments were conducted at 293 K using 3 mm NMR tubes (Norell). A standard ^1^H 1D presaturation experiment (zgpr) and the first increment of 1D NOESY (noesygppr1d) experiment were recorded and processed for both hydrophilic and lipophilic samples as previously reported [24]. All data were collected using a spectral width of 20.0 ppm, 64K points resulting in an acquisition time of 1.93 s, and on-resonance presaturation for solvent suppression during a 4 s recycle delay. The zgpr experiment was used to screen samples and check shimming. For hydrophilic samples, 1D NOESY spectra were collected with 64 scans, 4 dummy scans, 10 ms mixing time, and presaturation at the residual water frequency. The 90° pulse widths, measured for each sample using the automatic pulse calculation experiment (pulsecal) in TopSpin 2.1.1 (Bruker BioSpin, Billerica, MA), were between 8.3 and 8.8 µs. For lipophilic samples, first increments of 1D NOESY spectra were collected with 128 scans, 4 dummy scans and an offset frequency of 6194.14 Hz (7.36 ppm) in order to saturate the signal from residual chloroform. The 90° pulse widths for the lipophilic samples were between 7.7 and 8.1 µs.

### Multivariate statistical analysis of NMR spectra

Principal components analyses (PCA) and statistical significance analyses were performed using AMIX (Bruker BioSpin, Billerica, MA). Only first increments of 1D NOESY spectra were used for PCA. The numbers of cells were counted in each culture dish prior to trypsin treatment. Two different spectral normalization techniques were tested including normalization to total intensity and normalization to the total cell count. We assessed the performance of the two methods by evaluating the cluster spread of replicate samples in a PCA scores plot. We found that normalization to total intensity produced tighter clustering compared to normalization by total cell count. Therefore, the normalization to total intensity technique was used and all spectra were normalized to total intensity prior to PCA. An internal TSP standard of known concentration (580 µM) was included in all samples. Analysis of the TSP concentrations following normalization indicated that the difference in the TSP concentrations among replicates of the same cell line group, or between replicates of different cell line groups, was not statistically significant (*p* > 10^−1^ for all TSP concentration comparisons). This analysis indicated that the normalization procedure did not introduce significant systematic error into the peak intensities. For PCA, the Advanced Bucketing feature in Amix was used and up to five principal components had to be considered in order to account for 95% of the variance in the data (Table 2). Peaks that experienced less than 5% variance were excluded from PCA to simplify data analysis. The bucket width was optimized to match the width of the resonances at the base of the peaks, so that the integrated bucket intensities reported accurately on the integrated peak areas. Spectra were binned into 0.005 (hydrophilic) and 0.03 (lipophilic) ppm wide buckets over the region 0.01 – 10.0 ppm. A critical value (α-value) of 0.05 was selected to ensure a false-positive rate of no more than 5% in determination of significant changes in metabolite concentrations following treatment based on p-score evaluation. In order to maintain a constant family-wise error, and to correct for making multiple simultaneous inferences from each data set, a Bonferroni correction was applied by dividing α by the number of buckets used in the PCA. The Bonferroni-corrected α-values using 0.005 ppm buckets for hydrophilic cell extracts, determined for each comparison, are listed in Table 2. The Bonferroni-corrected α-values using 0.03 ppm buckets for lipophilic cell extracts were determined for each comparison and are also listed in Table 2. Changes in metabolite concentrations with corresponding *p*-values less than the Bonferroni-corrected α-value were considered statistically significant. The loading plot data points were color-coded according to bucket *p*-values: Black (>α-value, i.e. not statistically significant), Blue (α-value >10^−5^), Green (10^−5^ – 10^−6^), Yellow (10^−6^ – 10^−7^), Red (10^−7^ – 0). One-dimensional heat-map plots were generated using Microsoft Excel as described previously [25]. Mean differences for bucket intensities between groups were calculated by subtracting one group of normalized bucket intensity means from another group of normalized bucket intensity means. Fold-changes were calculated by dividing the treated bucket means by the control bucket means. For fold-changes less than 1.0, the negative inverse of the treated to control ratios were reported and the resulting values were referred to as “negative” fold-changes.

### Identification of metabolites

The statistical significance analysis described by Goodpaster et al. [25] was used to identify bucket frequencies that showed statistically significant changes in intensity in comparisons between cell lines. Metabolites corresponding to these resonances were then identified using chemical shift assignments of spectra of hydrophilic cell extracts based on comparison with chemical shifts of metabolites in Biological Magnetic Resonance Data Bank (BMRB) (http://www.bmrb.wisc.edu/metabolomics/), in the ChenomX NMR Suite (ChenomX Inc., Edmonton, Alberta, Canada) and in other published data [18,26–28]. For some tentatively assigned metabolites, an authentic standard sample was purchased (Sigma Aldrich), prepared in the hydrophilic buffer, and NMR spectra collected as above for comparison of chemical shifts and confirmation of tentative assignments. Chemical shifts of resonances in lipophilic extracts were compared against the chemical shifts of resonances of known metabolites in the BMRB and other published data [18,19,23,29].

### Determination of metabolite concentrations

Concentrations of metabolites for each treatment group were determined in each replicate using the ChenomX NMR Suite software package (http://www.chenomx.com/) by quantitative comparison of metabolite peak intensities relative to that of the TSP added to each sample as both a chemical shift and concentration reference standard. Specifically, the ChenomX software was used to adjust the intensity of the NMR spectrum of the metabolite stored in the reference database until it matched the intensity of the peaks in the experimental spectrum, and concentrations of the metabolites were measured relative to the internal TSP standard of known concentration, in this case, 580 mM. Since the concentration measurements were based on quantification relative to an internal standard, the concentration determinations were independent of any potential normalization artifacts. Only non-overlapped peaks were used to determine concentrations for each metabolite. For metabolites containing multiple non-overlapped peaks, the means and standard deviations of the concentrations were calculated using all non-overlapped peaks and using all replicate data.

## Results and Discussion

### NMR data and spectral assignments

**Figure 1:**
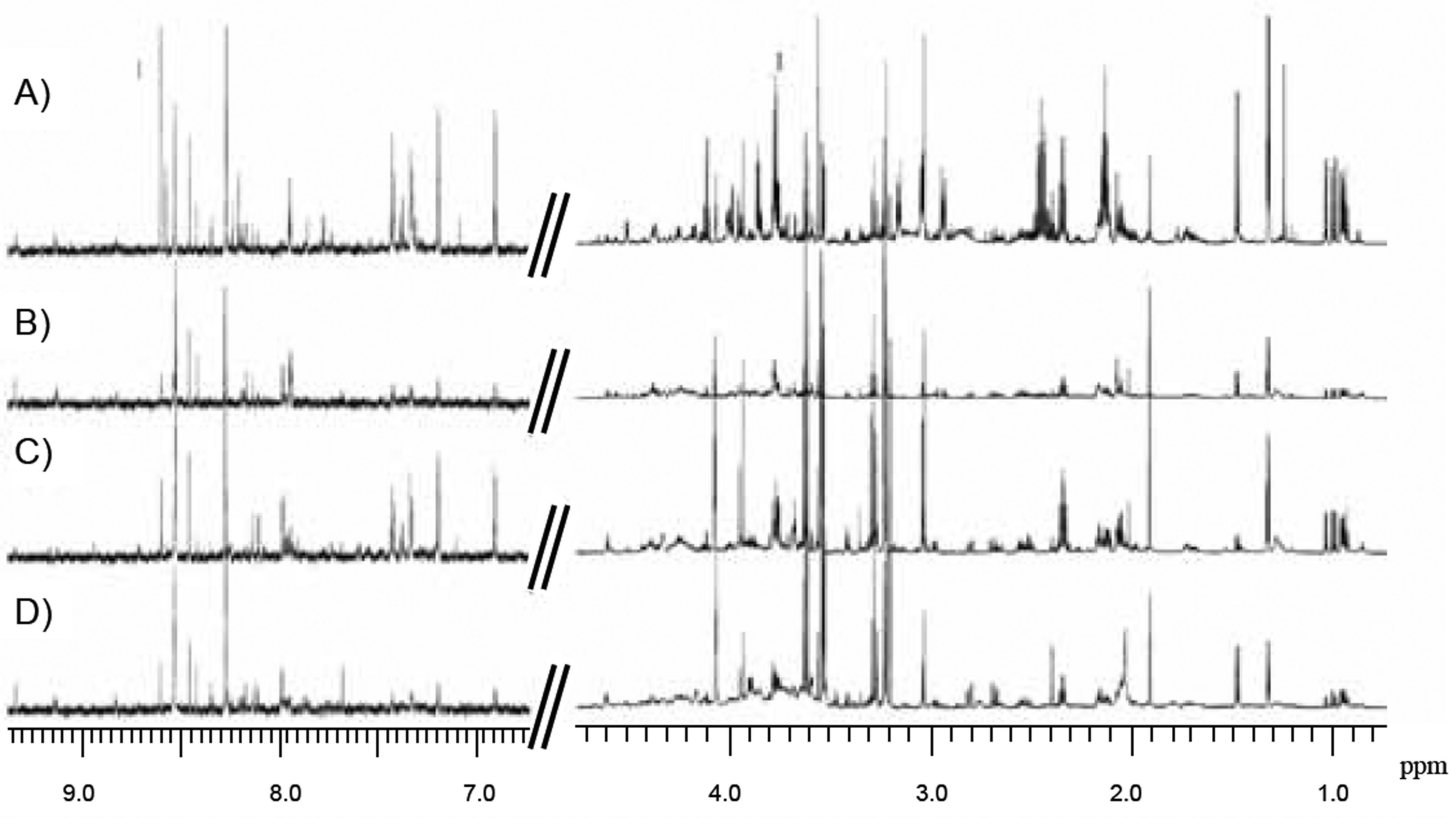
Representative one-dimensional 850 MHz 1H NMR spectra of hydrophilic extracts of each cell line. A) H6C7, B) Miapaca-2, C) Panc-1, D) AsPC-1. Spectral range displayed is 0.75 – 4.75 ppm and 6.5 −9.70 ppm.

**Figure 2:**
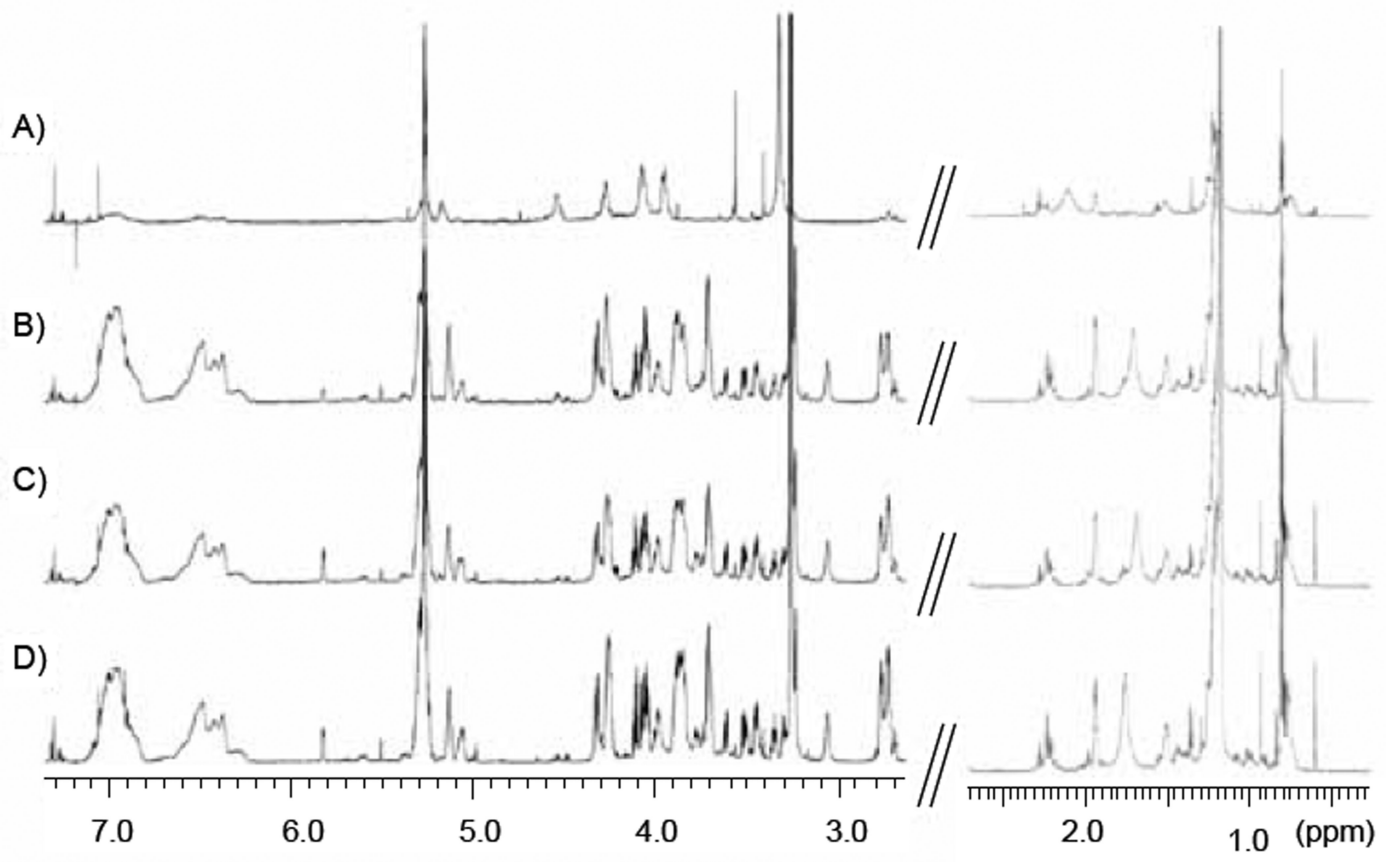
Representative one-dimensional 850 MHz 1H NMR spectra of lipophilic extracts of each cell line. A) H6C7, B) Miapaca-2, C) Panc-1, D) AsPC-1. Spectral range displayed is 0.35 – 2.65 ppm and 2.65 −7.30 ppm.

NMR data were collected on six, or seven in the case of H6C7, replicate samples of hydrophilic and lipophilic cell extracts from each cell line: H6C7, Miapaca-2, Panc-1 and AsPC-1. Representative spectra of hydrophilic and lipophilic extracts of each cell line are shown in Figures 1&2, respectively. Expected water-soluble cell metabolites such as amino acids, organic acids and bases were observed in spectra of hydrophilic extract samples and partial assignments are listed in Table 3. Water-insoluble metabolites such as fatty acids and cholesterol were observed in spectra of lipophilic extract samples and partial assignments are listed in Table 4.

### Unsupervised principal component analysis (PCA)

**Figure 3:**
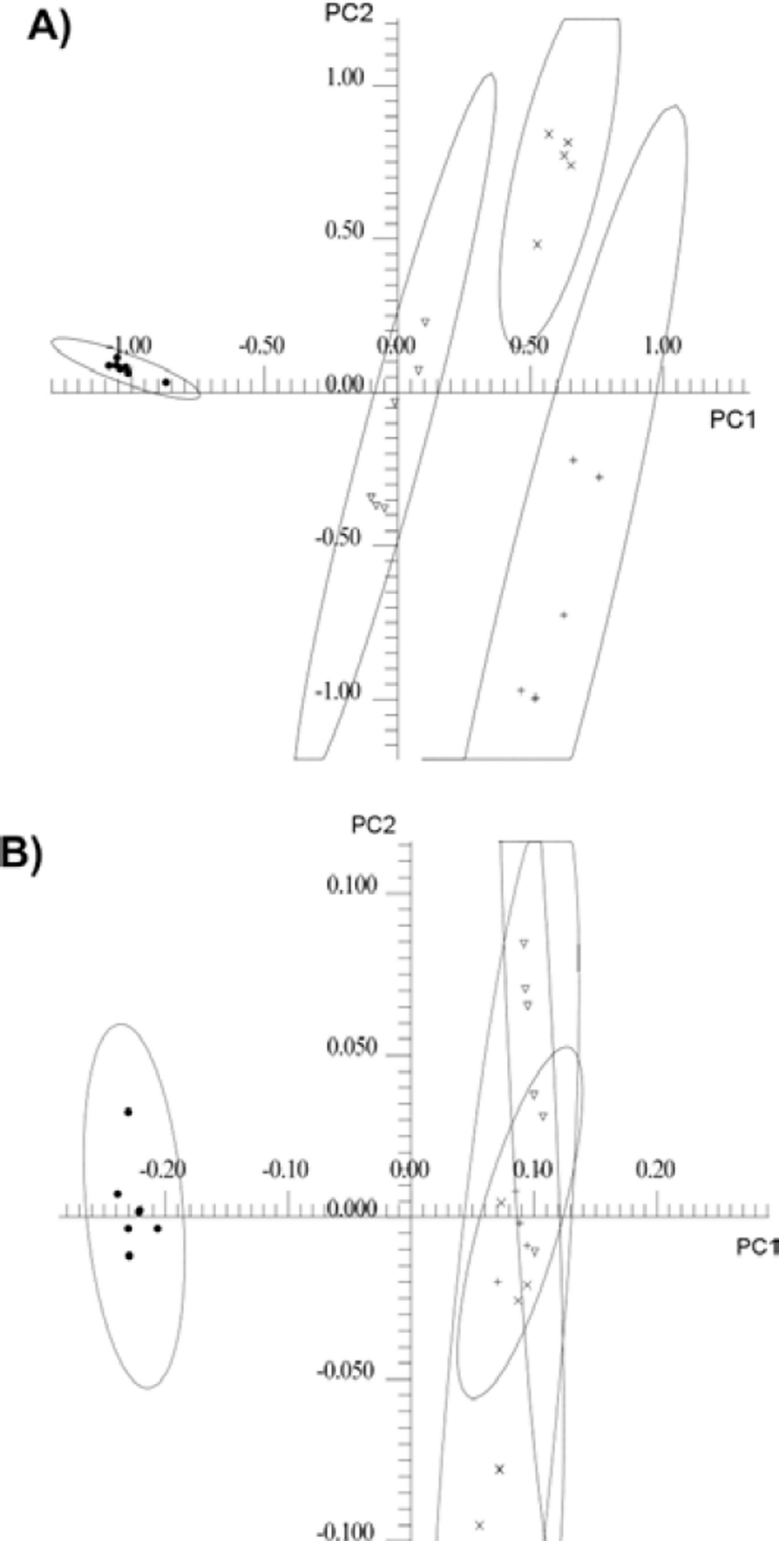
(A) Two-dimensional PC1 versus PC2 scores plot of hydrophilic cell extract data. •) H6C7, ▼) Miapaca-2, x) Panc-1, and +) AsPC-1. The 95% confidence interval for the four clusters of data points were indicated with oval lines. (B) Two-dimensional PC1 versus PC2 scores plot of lipophilic cell with the 95% confidence intervals indicated with oval lines.

In order to obtain a global sense of how the metabolic profiles of the cell lines compared, an unsupervised PCA was carried out on the 25 normalized 1D ^1^H-NMR spectra obtained from hydrophilic cell extracts from all four cell lines. The PCA scores plot of the hydrophilic cell extracts using the first two PCs showed complete separation of all cell lines at the 95% confidence intervals (Figure 3A). The loadings plot corresponding to the scores plot for the hydrophilic cell extracts is shown in Figure S1A. The lipophilic extract data was analyzed in a similar manner. Distinct separation was found between normal pancreatic ductal epithelial cells and pancreatic cancer cells however no distinct separation was observed among the cancer cell lines (Figure 3B). The loadings plot corresponding to the scores plot for the lipophilic cell extracts is shown in Figure S1 B.

### Pair-wise PCA and statistical significant analysis of hydrophilic cell extracts

**Figure 4:**
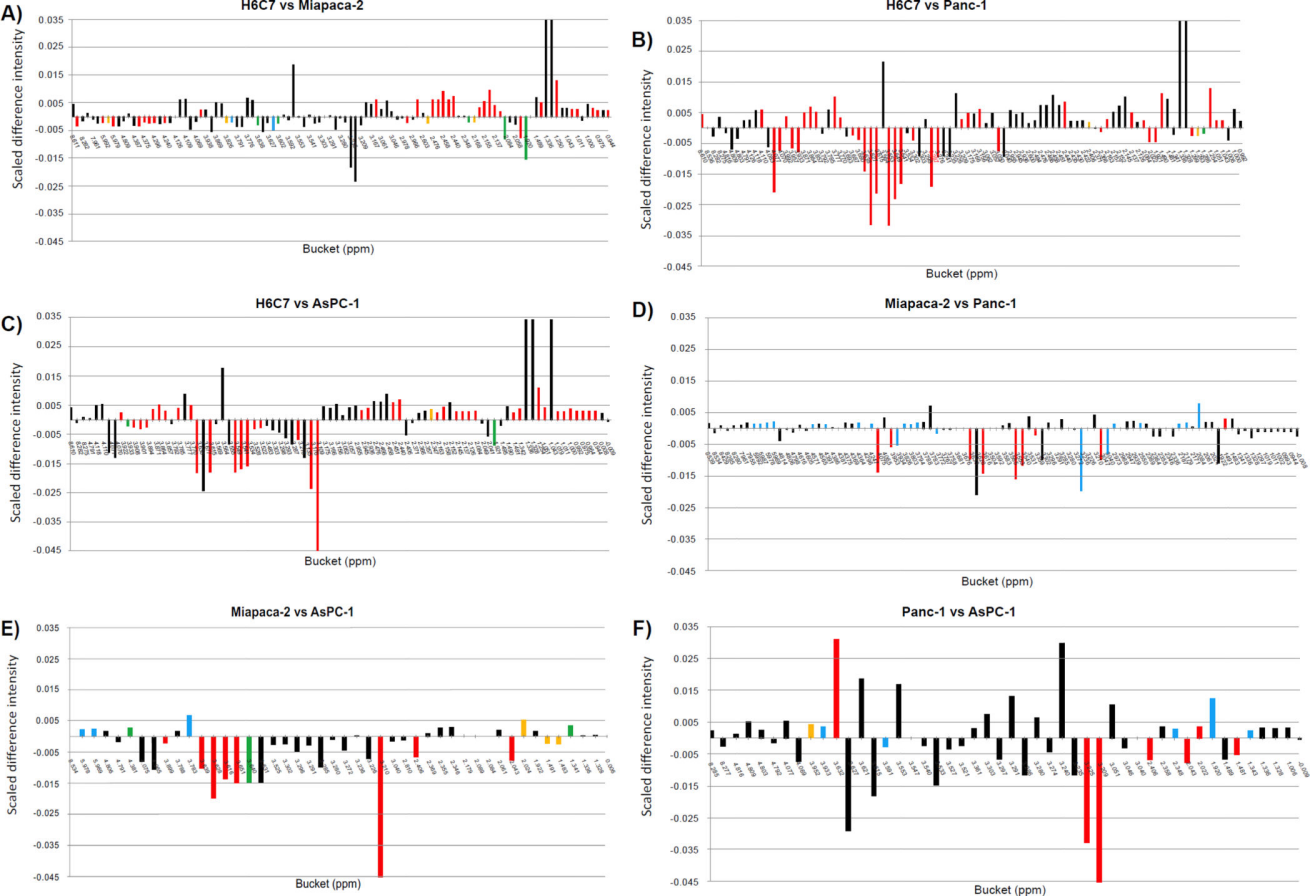
Heat-map color-coding of one-dimensional plot of mean differences calculated for bucket intensities for hydrophilic extracts; A) H6C7 and Miapaca-2, B) H6C7 and Panc-1, C) H6C7 and AsPC-1, D) Miapaca-2 and Panc-1, E) Miapaca-2 and AsPC-1, F) Panc-1 and AsPC-1. Scaled difference intensities were calculated by subtracting one normalized mean value from another. The heat maps are color-coded according to bucket p-values: Black (>α-value), Blue (α-value – 10-5), Green (10-5- 10-6), Yellow (10-6 – 10-7), Red (10-7-0).

Six pair-wise PCA comparisons were conducted, including H6C7 to Miapaca-2, H6C7 to Panc-1, H6C7 to AsPC-1, Miapaca-2 to Panc-1, Miaapaca-2 to AsPC-1, and Panc-1 to AsPC-1. Scores plots and loadings plots of each analysis are shown in Supplementary Figure S2. All comparisons indicated complete group discrimination and separation of the groups into distinct clusters at the 95% confidence intervals. All buckets with significant changes and their corresponding *p*-values are listed in (Table 3). One-dimensional heat-map plots for the same comparisons (Figure 4) show how the significant buckets were distributed across the spectra. Globally, similar numbers of significant buckets, (ranging from 41 to 43, (Table 2) were found in the comparisons between H6C7 cells and each of the cancer cell lines (Figure 4A–C). Very similar patterns of positive mean differences of bucket intensities in the aliphatic chemical shift range, around 0.00 to 3.17 ppm, were observed in these comparisons. Fewer significant buckets were detected ranging from 14 to 30, (Table 2) in the comparisons between cancer cell lines (Figure 4D–F).

### Pair-wise PCA and statistical significant analysis of lipophilic cell extracts

**Figure 5:**
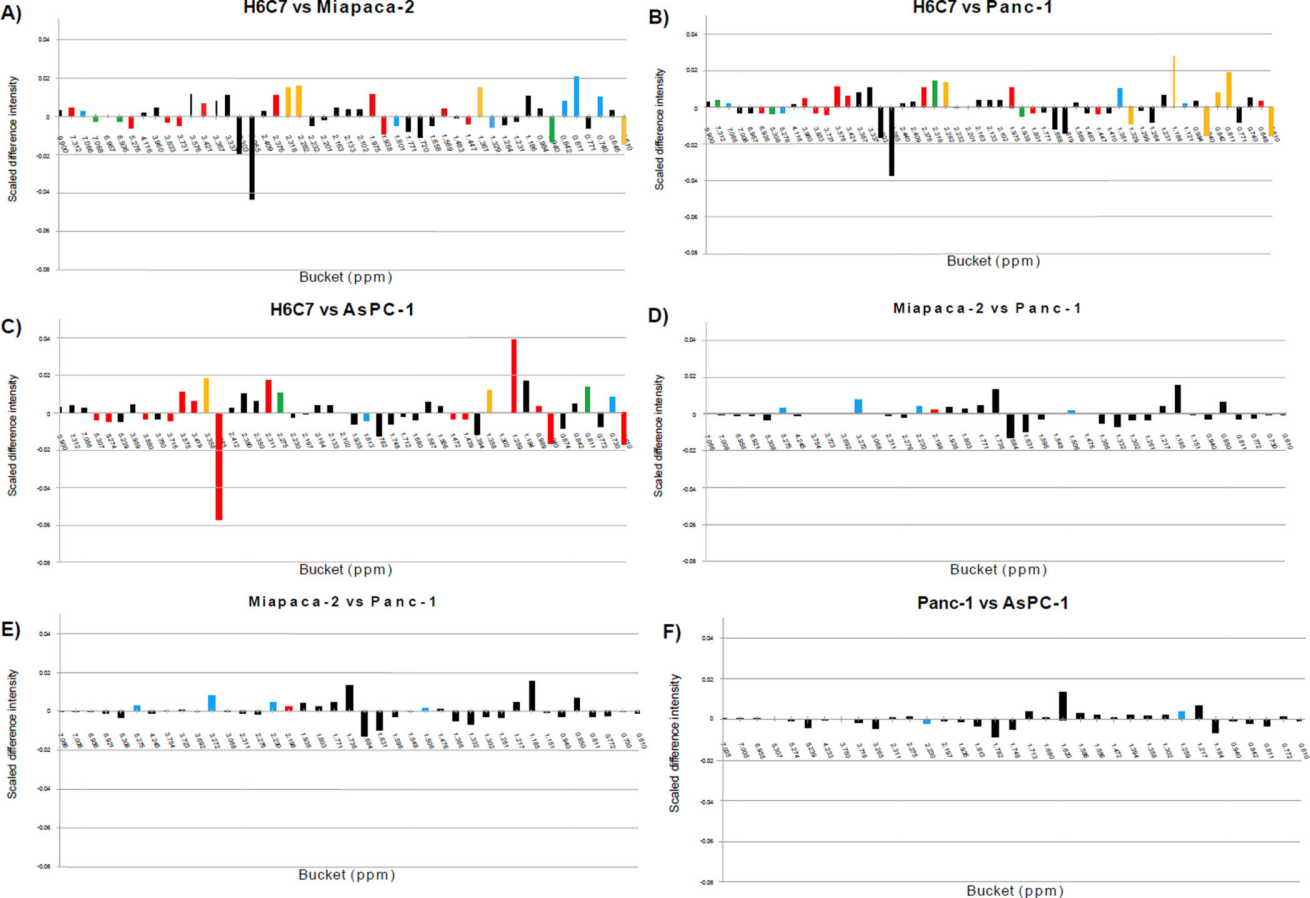
Heat-map color-coding of one-dimensional mean difference plots calculated for bucket intensities for lipophilic extracts; A) H6C7 and Miapaca-2, B) H6C7 and Panc-1, C) H6C7 and AsPC-1, D) Miapaca-2 and Panc-1, E) Miapaca-2 and AsPC-1, F) Panc-1 and AsPC-1. Scaled difference intensity was calculated by subtracting one normalized mean value from another. The heat maps are color-coded according to bucket p-values: Black (>α-value), Blue (α-value – 10-5), Green (10-5- 10-6), Yellow (10-6 – 10-7), Red (10-7- 0).

PCA scores plots for pair-wise comparisons of lipophilic cell extracts showed complete separation between H6C7 and all cancer cell line extracts (Supplementary Figure S3). Pair-wise PCA on lipophilic extracts of different cancer cells showed no separation between cancer cell lines (Supplementary Figure S3 D – F). One-dimensional heat-maps showed similar patterns of significant buckets in the comparisons between each cancer cell line and the normal pancreatic cells (Figure 5A–C). Few significant buckets were found in comparisons between cancer cells (Figure 5D–F). All significant buckets and their corresponding *p*-values are listed in (Table 4).

### Identification of metabolites and determination of fold-changes

Over 25 hydrophilic metabolites were identified as causing separation between healthy and cancer cell lines in PCA scores plots. Metabolites identities along with *p*-values and fold-changes for each significant bucket are summarized in (Table 3&4). Buckets that contained resonances from more than one metabolite were eliminated from this table. Peaks that clearly belonged to the same metabolite but experienced some peak shifting between cell line extracts were aligned prior to statistical significance analysis. This was a particular problem for peaks belonging to myo-inositol, which had clustered multiplets that experienced slight shifts between cell line extracts. In order to calculate *p*-values for myo-inositol peaks, wider 0.01 ppm buckets were used to measure the integrated intensity of the entire multiplet structures and the peaks were then aligned prior to integration. Once the metabolite identities were confirmed, the concentrations of the metabolites were measured relative to the internal TSP standard. The average and standard deviations of the concentrations of most of the hydrophilic metabolites along with the fold-changes based on concentration measurements are reported in (Table 5). The fold-changes obtained from changes in peak intensities and concentration estimations were consistent. It was impossible to calculate reliable fold-changes when metabolites were absent or nearly absent in one of the cell lines being compared (e.g. acetamide and glutathione).

Although it was challenging to assign metabolites in lipophilic extracts due to lack of complete databases, some cellular membrane components such as lipids and cholesterol were identified (Table 4). By comparison with data from previous literature, singlets at 0.61 and 0.94 ppm were assigned to C19 and C18 cholesterols and multiple features from fatty acids were identified.[19,23,29] The presence of phospholipids was confirmed by comparing spectra to standards of phosphatidylcholine and phosphatidylethanolamine, which had expected singlets at 3.35 and 3.27 ppm, respectively.

### Differences in metabolite concentrations between H6C7 and all cancer cells

**Figure 6:**
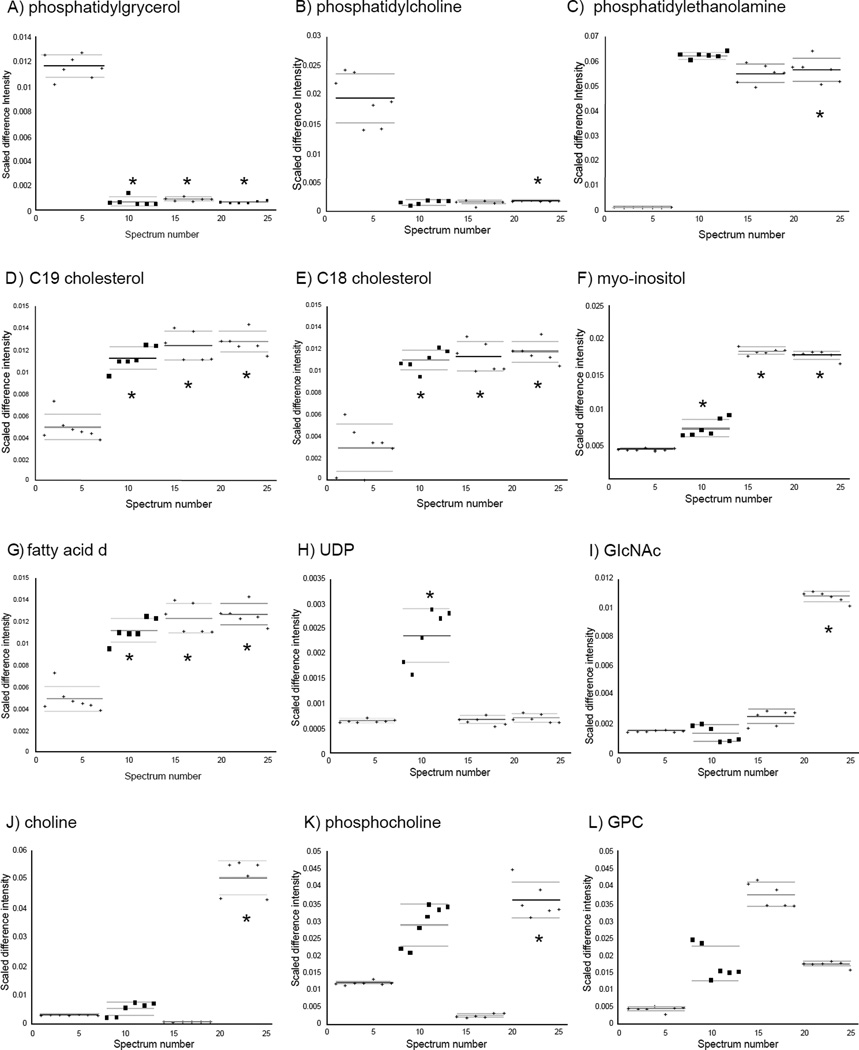
Plots showing distributions of normalized data points for the 12 metabolites with significant changes involved in cellular membrane composition. From left to right, H6C7, Miapaca-2, Panc-1, and AsPC-1. Solid lines indicate the group means and dashed lines indicate the 95% confidence intervals. (*) indicates p-values for comparison with the control group that are smaller than Bonferroni-corrected a-values (The p-values are listed in Table 2). A) phosphatidylgrycerol, B) phophatidylcholine, C) phosphatidylethanolamine, D) C19 cholesterol, E) C18 cholesterol, F) myo-inositol, G) fatty acid δ, H) UDP, I) GlcNAc, J) choline, K) phosphocholine, L) GPC.

**Figure 7:**
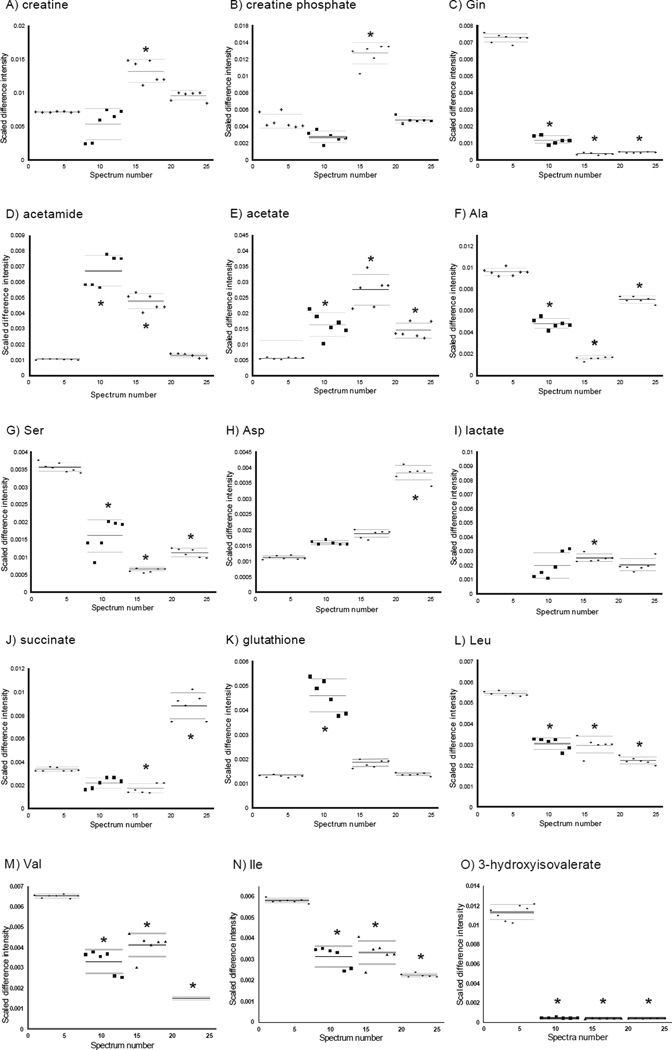
Plots showing distributions of normalized data points for the 15 metabolites with significant changes involved in creatine pathway, glutamine metabolism and protein synthesis. From left to right, H6C7, Miapaca-2, Panc-1, and AsPC-1. Solid lines indicate the group means and dashed lines indicate the 95% confidence intervals. (*) indicates p-values for comparison with the control group that are smaller than Bonferroni-corrected a-values (The p-values are listed in Table 2). A) creatine, B) creatine phosphate, C) Gln, D) acetamide, E) acetate, F) Ala, G) Ser, H) Asp, I) lactate, J) succinate, K) glutathione, L) Leu, M) Val, N) Ile, O) 3-hydroxyisovalerate.

Overall, 15 metabolites were identified with significantly altered concentrations in all cancer cells. All three cancer cell lines showed significantly decreased or nearly absent concentrations of phosphatidylgrycerol, phosphatidylcholine (Figure 6A, B), Gln, Ala, Ser, lactate, Leu, Val, Ile, and 3-hydroxyisovalerate (Figure 7C,F,G,I,L–O). Significantly increased levels of phosphatidylethanoamine, C19 and C18 cholesterols, myo-inosital (Figure 6C–F), and acetate (Figure 7E) were a common feature in all cancer cells.

### Unique differences in metabolite concentrations between H6C7 and individual cancer cell lines

In the hydrophilic extracts, several unique metabolites with altered concentrations were identified in each of the cancer cell lines. Four metabolites were identified whose concentrations were only altered in AsPC-1 cells, including increased choline and N-acetylglucosamine (GlcNAc) (Figure 6I,J), and decreased Glu, Asp, and succinate (Figure 7C,H,J). Increases in glutathione and uridine diphosphate (UDP) species were found in Miapaca-2 cells (Figure 6H,7K) and increases in creatine, creatine phosphate, and octanoic acid were only detected in Panc-1 cells (Figure 7A,B). In addition, increases in acetamide were found in Miapaca-2 and Panc-1 cells but not in AsPC-1 cell (Figure 7D).

### Differences in metabolites concentrations that indicate differences in cellular membrane composition between H6C7 and cancer cells

Altered cellular membrane composition in pancreatic cancer cells compared to non-cancerous ductal epithelial cells was indicated by consistently differential levels of metabolites involved in glycerophospholipid metabolism, lipopolysaccharide biosynthesis and fatty acid biosynthesis. The major components of the mammalian cell membrane are phosphatidylcholine, phosphatidylethanolamine, phosphatidylinositol and phosphatidylgrycerol.

### Glycerophospholipid metabolism

Alteration in glycerophospholipid metabolism was supported by findings of increased phosphatidylethanolamine (all >10 fold, p <10^−2^, <10^−2^ and <10^−11^ for Miapaca-2, Panc-1 and AsPC-1, respectively), and phosphatidylgrycerol (all >10 fold, p <10^−11^, <10^−14^ and <10^−15^ for Miapaca-2, Panc-1 and AsPC-1, respectively), and decreased phosphatidylcholine (<-10 fold, *p* <10^−2^, −6.22 fold, *p* <10^−1^ and < −10 fold, *p* <10^−6^ for Miapaca-2, Panc-1 and AsPC-1, respectively) in lipophilic extracts of all cancer cell lines compared to H6C7 cells (Figure 6A–C). An increase in phosphatidylethanolamine in cell membranes has been correlated with increased membrane viscosity compared to phosphatidylcholine due to its polar head group [30]. Though a change in phosphatidylinositol was not observed in this study, increased myo-inositol was found in all three cancer cell lines (>1.55 fold, *p* <10^−4^, >1.43 fold, *p* <10^−11^, and >3.44 fold, *p* <10^−8^ for Miapaca-2, Panc-1 and AsPC-1, respectively) (Figure 6F). Since cytosine and myo-inositol are converted into phosphatidylinositol by phosphatidylinositol synthase, the change in myo-inositol levels may also indicate a change in phosphatidylinositol levels in the pancreatic cancer cells. In addition, altered levels of choline (1.72 fold, *p* <0.05, −3.52 fold, *p* <10^−8^, and >10 fold, *p* <10^−10^ for Miapaca-2, Panc-1 and AsPC-1, respectively), phosphocholine (2.62 fold, *p* <10^−2^, −4.52 fold, *p* <10^−1^, and 2.88, fold *p* <10^−2^ for Miapaca-2, Panc-1 and AsPC-1, respectively), and glycerophosphocholine (GPC) (4.35 fold, *p* <10^−2^, 4.48 fold, *p* <10^0^, and 3.42 fold, *p* <0.05 for Miapaca-2, Panc-1 and AsPC-1, respectively) in hydrophilic cell extracts strongly supports altered glycerophospholipid metabolism in pancreatic cancer cells compared to H6C7 cells (Figure 6 J–L). Our observations of altered lipid and fatty acid composition in these pancreatic cancer cells is consistent with patterns of altered cell membrane components identified in other cancer cells [31–35]. For example, alterations in major classes of lipids, e.g. phosphatidylcholine, phosphatidylethanolamine, phosphatidylserine, sphingomyelin and phosphatidylinositol, have also been found in breast cancer cells [36]. In addition, further studies have shown that tumor cell membranes have higher resistance to transitions to non-lamellar phases, which is associated with a decrease in membrane permeability [36]. Even though no common pattern in the changes in lipid compositions of cancer cells has been identified, phospholipid compositions have been suggested as a potential method for distinguishing between benign and malignant types of tumors [37]. Metabonomics studies of plasma lipids have also demonstrated the potential use of lipid alterations as biomarkers for pancreatic cancer detection [11].

### Cholesterols

Another indication of a distinct cellular membrane composition in the pancreatic cancer cells was related to apparently high cholesterol to phospholipid ratio in the pancreatic cancer cells. The molar ratio of cholesterol to phospholipid in normal eukaryotic cellular membranes is 0.8 to 0.9 in platelets [38] and this ratio is known to be altered by the availability of cellular cholesterol (reviewed in Specto, et al.) [30]. Here, more than two-fold increases in androgens (C19 cholesterol), (2.29 fold, *p* <10^−6^, 2.58 fold *p* <10^−6^, and 2.52 fold, *p* = <10^−7^ for Miapaca-2, Panc-1 and AsPC-1 respectively), and estrogens (C18 cholesterol) (2.86 fold, *p* <10^−6^, 3.01 fold, *p* <10^−6^, and 2.95 fold, *p* <10^−7^ for Miapaca-2, Panc-1 and AsPC-1, respectively), were identified in lipophilic extracts of all cancer cells compared to normal cells (Figure 6 D, E). The dysfunction or absence of a cholesterol feedback control mechanism has been identified in hepatic cancers and leukemic cells [39]. The absence of feedback results in increased levels of cholesterol in cancer cells and has also been reported in breast cancer patients [40]. The majority of cellular cholesterols are embedded in the membranes thereby affecting permeability of the plasma membrane, function in intracellular transport, and cell signaling. Increases in cholesterol levels support the hypothesis of altered membrane composition in cancer cells compared to normal cells.

### Fatty acid saturation

Altered ratios of saturated versus unsaturated fatty acids in pancreatic cancer cells in comparison to healthy ductal epithelial cells were also suggested from our data. Though the exact identification of specific fatty acids in lipophilic extracts was not possible, changes in peaks from fatty acids were observed. For example, increased intensities in 5.276 ppm (1.59 fold, *p* <10^−8^, 1.30 fold, *p* <10^−4^, and 1.43 fold, *p* <10^−7^ for Miapaca-2, Panc-1 and AsPC-1, respectively) (Figure 6G) and 5.307 ppm (1.73 fold, *p* <10^−5^, and 1.72 fold, *p* <10^−9^ for Panc-1 and AsPC-1, respectively) buckets, which belong to -CH=CH-of fatty acids, were observed in cancer cell lines compared to normal cells. On the other hand, decreases in bucket intensities at 1.121 ppm (−1.63 fold, *p* <10^−3^, and −2.26 fold, *p* < 10^−10^ for Panc-1 and AsPC-1, respectively), and 1.186 ppm (−1.04 fold, *p* <10^−1^, −1.11 fold, *p* <10^−6^, and −1.07 fold, *p* <10^−1^ for Miapaca-2, Panc-1 and AsPC-1, respectively), which belong to -CH_2_-CH_2_-CH_2_- of fatty acids, were found in cancer cell lines. These observations indicated increased abundance of polyunsaturated chains in cancer cells compared to normal cells. Several studies of tumor metabolism have demonstrated changes in lipid metabolism (reviewed in Griffin et al.) [5]. Our observations are consistent with observations of higher levels of unsaturated fatty acids in cancer cell lines in a comparison between a breast cancer metastatic cell line M-4A4 and a nonmetastatic cell line NM-2C5 [41]. In addition, higher expression of fatty acid desaturase, an enzyme which converts saturated fatty acids to unsaturated fatty acids by inserting double bonds, has been also shown in M-4A4 [42]. The treatment of hepatic cells with monounsaturated oleic acid has been shown to promote cell proliferation by inhibiting expression of tumor suppressor phosphatase and tensin homolog [43,44].

### Evidence of differences in cellular membrane composition between cancer cell lines

Evidence of differences in cellular membrane compositions among three pancreatic cancer cell lines were also apparent from pair-wise comparisons between hydrophilic extracts of cancer cell lines. For example, in Miapaca-2 cells, significant increases in two doublets at 5.97 ppm (4.75 fold, *p* <10^−7^), and 5.99 ppm (3.91 fold, *p* <10^−6^) were observed (Figure 6H). These resonances were identified as belonging to UDP species such as UDP, UDP-GlcNAc, and UDP N-acetylgalactosamine (GalNAc). These UDP-relasted metabolites are involved in lipopolysaccharide biosynthesis. The concentration of UDP-GlcNAc has been shown to be an important factor in the biosynthesis of beta 1,6-branched oligosaccharides, which have increased levels in many malignant tumors [45]. Increased octanoic acid levels (>4 fold, *p* <10^−4^) were found in Panc-1 cellular extracts. Octanoic acid is involved fatty acid biosynthesis, which, together with changes in UDP, suggests differences in cellular membrane compositions between different cancer cell lines.

In hydrophilic AsPC-1 cell extracts, a unique feature compared to other cell lines was found in the region from 2.0 to 2.1 ppm (Figure 6I). Based on the literature, this broad feature was assigned to the N-acetyl groups of mobile carbohydrate side-chains such as GlcNAc and N-acetylneuraminic acid [46,47]. Increased intensities of this resonance have been identified in ovarian tumor tissue and shown that it was mainly derived from the methyl group of N-acetylaspartic acid (NAA) [46]. Increased levels of glycolipids were also found in TL1049 and TL3544 testicle cancer cell lines [48]. Our results suggest that AsPC-1 has higher levels of N-acetyl glycoproteins and/or glycolipids compared to other cell lines, H6C7, Miapaca-2, and Panc-1 (5.21 fold, *p* < 10^−1^, 5.47 fold, *p* < 10^−1^, and 7.38 fold, *p* < 10^−14^, respectively). Since glycolipids and glycoproteins are mainly found in the outer layer of the cellular membrane, changes in these metabolites strongly support the alteration of membrane compositions in AsPC-1 cells. In addition, since NAA is converted into aspartate and acetate by aspartoacylase, increased level of aspartate in AsPC-1 cells also supports the hypothesis of a difference in cellular membrane compositions among three pancreatic cancer cell lines.

These data strongly suggests the differences in cellular membrane composition present not only between cancer cells and normal cells but also between cancer cell lines. Collectively, the inferred changes in fatty acid compositions in pancreatic cancer cells indicate alterations of membrane fluidity, membrane composition, signal transduction pathways through the membrane, and gene expression or activities. Alternatively, these changes could affect the rate of cell growth and cell death [49].

### Differences in creatine pathway activity among cell types

Increased creatine (1.89 fold, *p* <10^−7^) and creatine phosphate (2.77 fold, *p* <10^−7^) levels were observed hydrophilic extracts of Panc-1 cells (Figure 7A,B). In the presence of ATP, creatine is converted into phosphocreatine by creatine kinase, and when cells require high energy, ATP is synthesized from ADP and phosphocreatine. Increased activity of creatine kinase isoenzyme BB, which is stimulated by hormones or growth factors, has been shown in breast cancer cell lines CG5 and MCF-7 [50]. Increased levels of creatine are associated with increased protein synthesis. Induced creatine metabolism suggests increased creatine kinase activity in pancreatic cancer cell lines and could be a potential characteristic of cancer cells. An induced creatine pathway in cancer cell lines was also suggested by the observation of decreased levels of glycine in all three cancer cell lines. Further study is required to determine if increased creatine kinase expression is involved in an induced creatine pathway in pancreatic cancer cell lines.

### Differences in glutamine metabolism in pancreatic cancer cells compared to H6C7 cells

A dramatic decrease or absence of intracellular glutamine levels was detected in all three pancreatic cancer cell lines (−5.44 fold, *p* <10^−11^, < −10 fold, *p* <10^−14^, and < −10 fold, *p* <10^−13^ for Miapaca-2, Panc-1 and AsPC-1, respectively) compared to H6C7 (Figure 7C). Changes in other metabolites involved in glutamine metabolism and the TCA cycle were observed such as an increase in acetamide (6.99 fold, *p* <10^−13^ and 4.52 fold, *p* =10^−10^ for Miapaca-2, and Panc-1, respectively) and acetate, (3.23 fold, *p* <10^−4^, 4.90 fold, *p* <10^−6^, and 2.38 fold, *p* <10^−5^ for Miapaca-2, Panc-1 and AsPC-1 respectively), and a decrease in alanine (−1.77 fold, *p* <10^−7^, < −10 fold, *p* <10^−14^, and −1.42 fold, *p* <10^−8^ for Miapaca-2, Panc-1 and AsPC-1 respectively), lactate (−4.26 fold, *p* <10^−3^, −3.30, *p* <10^−9^, and −4.15 fold, *p* <10^−3^ for Miapaca-2, Panc-1 and AsPC-1 respectively) and serine (−2.07 fold, *p* <10^−8^, −5.84 fold, *p* =10^−14^, and −3.48 fold, *p* <10^−12^ for Miapaca-2, Panc-1 and AsPC-1 respectively) in the cancer cells (Figure 7D–G,I).

### Pancreatic cancer cell line-specific differences in glutamine metabolism

Some cell line specific changes, which related to glutamine metabolism, were also found in hydrophilic extracts. A decrease in Glu (−2.06 fold, *p* <10^−7^) and an increase in Asp (3.36 fold, *p* <10^−13^) and succinate (2.42 fold, *p* <10^−7^) were found in AsPC-1 cells (Figure 7H,J). In addition, an increase in glutathione (>3.09 fold, *p* <10^−5^) was observed in Miapaca-2 cells (Figure 7K). Glutamine has been recognized for a long time as one of the major energy sources for tumor cells in its role as a substrate for mitochondrial oxidation [51,52]. The increased activity of glutaminase, which converts glutamine to glutamate as the first step in metabolic intermediate production required in cell growth, has been previously reported in cancer cells [52,53]. Low intracellular levels of glutamine has been an indicator for induced nucleotide biosynthesis, protein synthesis and cell growth in tumor cells [53]. Currently, glutaminolysis, a mechanism in which glutamine is converted into lactate, is considered a common characteristic of tumor cell metabolism (reviewed in Deberardinis et al.) [54].

### Indication of altered protein synthesis activity in all pancreatic cancer cell lines

In addition to induced glutamine metabolism, changes in cellular levels of amino acids were found in all pancreatic cancer cells when compared to H6C7 cells, indicating induced protein synthesis. Decreases in Val (−1.95 fold, *p* <10^−7^, −1.60 fold, *p* <10^−6^, and −3.92 fold, *p* <10^−16^ for Miapaca-2, Panc-1 and AsPC-1, respectively), Ile (−1.63 fold, *p* <10^−5^, and >-2.51 fold, *p* <10^−13^ for Miapaca-2 and AsPC-1, respectively), and Leu (−1.79 fold, *p* <10^−9^, and −2.36 fold *p* <10^−11^ for Miapaca-2, and AsPC-1, respectively) in pancreatic cancer cells could be caused by induction of protein synthesis that consumes these amino acids [55] or decreased synthesis or uptake of these amino acids (Figure 7 L–N). In fast proliferating cells, protein synthesis is known to be induced and is known to lead to a decrease in free amino acids.

In addition, absence or decreases in 3-hydroxyisovalerate (β-hydroxy β-methylbutyric acid) levels were found in all pancreatic cancer cells (all <-10 fold, *p* <10^−11^ for Miapaca-2, Panc-1 and AsPC-1) suggesting the presence of induced leucine metabolism (Figure 7O). The presence of 3-hydroxyisovaleric acid is an indicator of reduced activity of biotin-dependent enzymes in urine [56]. Increased 3-hydroxyisovaleric acid level is a consequence of incomplete leucine metabolism; thus, decreased levels of 3-hydroxyisovaleric acid may indicate induced Val, Ile, and Leu biosynthesis.

Increased levels of Thr in Miapaca-2 (3.62 fold, *p* <10^−4^) along with decreased serine levels were observed in the cancer cell lines and these changes are also consistent with induced protein synthesis because Gly, Ser and Thr metabolism is closely connected to, and precedes, Val, Ile and Leu biosynthesis.

## Conclusions

Here, metabolic profiles of normal pancreatic ductal epithelial cells, H6C7, and three pancreatic cancer cell lines, AspC-1, Panc-1, and Miapaca-2, were compared. Several metabolites involved in glycerophospholipid metabolism, lipopolysaccharide biosynthesis, fatty acid biosynthesis, and N-acetyl glycoprotein/glycolipids metabolism were different between all cancer cell types and non-cancerous H6C7 cells indicating differences in cellular membrane composition in highly proliferative cells. Increased levels of cholesterol and polysaturated fatty acids were also observed in all cancer cells, suggesting the presence of higher membrane fluidity in pancreatic cancer cells compared to normal cells. Evidence of induced glutamine metabolism was also found in all pancreatic cancer cell types indicated by absence of intercellular glutamine and changes in the abundance of several amino acids and TCA cycle intermediates. There were also metabolic differences identified between pancreatic cancer cell lines. For example, the presence of increased N-acetyl groups in AsPC-1, octanoic acids in Panc-1, and UDP species in Miapaca-2 strongly suggest the differences in cellular membrane composition between pancreatic cancer cell types. Cell line specific differences in metabolite concentrations may be potentially useful as an indicator for states of cell differentiation, invasiveness, growth rate, and origins of cells. A potential limitation of this study is the fact that only three different human pancreatic cancer cell lines were examined. Ideally, a larger number of human pancreatic cancer cell lines should be included in future investigations to ensure a broader spectrum of the metabolic profile of pancreatic cancer cells can be established. Further investigations are needed to determine the cause of the cell line specific changes in metabolite levels and to explore the potential value of pancreatic cancer cell-specific characteristic for future use in developing therapies or diagnoses.

## Supplementary Material

supplemental

## Figures and Tables

**Table 1:** A summary of characteristics of cell lines used in the study.

Cell line	Origin	Morphology	Est. doubling time	Ref.
Miapaca-2	primary adenocarcinoma tumor	epithelial cell	40 hrs	45
Panc-2	primary tumor in the pancreatic duct of an epithelioid carcinoma	epithelial cell	56 hrs	46
AsPC-1	pancreas ascites from an adenocarcinoma patient	epithelial cell	58 hrs	47
H6C7	pancreatic ductal epithelial cell			17

**Table 2:** Total number of buckets for each comparison (after excluding those with less than 5% variance), the number of significant buckets, and Bonferroni-corrected *a*-values of pair–wise PCA analysis for each comparison from hydrophilic and lipophilic extracts.

	Hydrophilic extract (0.005ppm)			Lipophilic extracts (0.03ppm)		
	Total number of buckets	Significant buckets	Bonferroni-corrected α-values	Total number of buckets	Significant buckets	Bonferroni-corrected α -values
**H6C7 – Miapaca-2**	250	42	4.76x10^−4^	48	24	1.04x10^−3^
- **Panc-1**	174	41	5.56x10^−4^	53	26	9.43x10^−4^
- **AsPC-1**	81	43	6.17x10^−4^	47	20	1.06x10^−3^
**Miapaca-2 – Panc-1**	84	30	5.95x10^−4^	39	5	1.28x10^−3^
- **AsPC-1**	54	14	9.26x10^−4^	39	4	1.28x10^−3^
**Panc-1 – AsPC-1**	49	14	1.02x10^−4^	35	0	1.43x10^−3^

**Table 3:** *P*-values and fold-changes for buckets associated with metabolites in hydrophilic cell extracts in the following comparisons: H6C7 and Miapaca-2, H6C7 and Panc-1, H6C7 and AsPC-1, Miapaca-2 and Panc-1 (MP), Miapaca-2 and AsPC-1 (MA), and Panc-1 and AsPC-1 (PA) are listed with the significant buckets highlighted. Some *p*-values were not reported for buckets experiencing less than 5% variance.

	Bucket	Miapaca-2	Fold	Panc-1	Fold	AsPC-1	Fold	MP	Fold	MA	Fold	PA	Fold
Metabolites	(ppm)	*P*-Value	change	*P*-Value	change	*P*-Value	change	*P*-Value	change	*P*-Value	change	*P*-Value	change
**Adenine /AMP**	8.611	2.70E-03	−5.30	2.04E-10	−4.40	5.54E-14	−5.81		***		***		***
**ATP/ADP**	8.538	6.00E-08	3.00	5.50E-02	2.73		***		***	6.44E-05	−1.98		***
**3-hydroxyisovaleric acid**	1.254	3.40E-12	<−10	5.74E-12	<−10	4.90E-12	<−10		***		***		***
**Acetamide**	2.023	6.05E-14	6.99	1.00E-10	4.52		***	4.70E-07	−1.54	7.32E-13	−5.97	6.65E-02	−3.86
**Acetate**	1.921	1.55E-05	3.23	6.79E-07	4.90	2.29E-06	2.38	1.02E-02	1.52	4.47E-02	−1.36	2.57E-04	−2.06
**N-acetlyglucosamine**	2.04		***		***	4.55E-02	5.21		***	5.47E-02	4.37	2.42E-15	7.38
**Alanine**	1.489	2.20E-01	−3.49	6.90E-15	<−10	2.70E-03	−2.06	9.12E-01	<−10	5.71E-01	1.94	4.84E-01	>10
	1.481	5.65E-08	−1.77	3.35E-02	<−10	3.23E-09	−1.42	6.92E-07	−3.19		***	2.51E-12	4.00
**Aspartate**	3.908		***		***	8.50E-14	3.36		***		***		***
	3.901		***		***	3.89E-13	4.73		***		***		***
	3.894		***		***	1.59E-13	3.51		***		***		***
**Choline**	3.209	3.17E-01	1.72	1.05E-09	−3.52	5.60E-11	>10	4.02E-02	−6.04	3.95E-03	8.50	3.45E-10	>10
**Creatine**	3.933	8.62E-01	>10	4.86E-08	1.89		***	8.51E-01	<−10	6.66E-01	1.37	1.05E-05	−1.51
	3.04	1.16E-01	1.07	5.50E-02	1.67		***	7.23E-06	1.58		***	2.76E-06	−1.62
**Creatine phosphate**	3.953		***	1.10E-08	2.77		***	7.36E-08	3.10		***	4.94E-07	−2.21
	3.046	1.52E-02	−1.55		***		***	6.12E-08	4.24		***	7.45E-01	>10
	3.053	2.70E-03	−6.14	3.09E-07	2.06	2.70E-03	−16.70		***		***	6.65E-02	−7.68
**Glutamate**	2.348	6.31E-01	−1.05		***	1.27E-08	−2.06	8.29E-01	−1.00	1.10E-03	<−10	3.35E-07	−1.94
	2.358	8.60E-01	1.01		***	2.70E-03	−1.89	6.66E-01	1.10	2.06E-04	−1.92	6.65E-02	−2.10
	*2.162	5.56E-13	−2.21	1.20E-07	−1.86	4.22E-13	−2.18	4.02E-02	−2.84		***		***
	*2.155	2.34E-12	−3.09	3.35E-02	−4.37	8.06E-13	−5.19		***		***		***
	*2.144	3.80E-14	−3.63	3.35E-02	−4.53	6.05E-15	−7.05		***		***		***
	*2.137	5.28E-11	−2.14	5.88E-12	−2.79	2.00E-13	−4.96		***		***		***
	*2.127	1.62E-08	−1.70	3.35E-02	−1.75	2.51E-12	−3.28		***		***		***
	*3.785		***	3.35E-02	−2.34	1.04E-10	−2.18		***	3.52E-06	−2.99		***
	*3.776	2.70E-03	−2.17	5.61E-13	−4.85	3.51E-08	−5.18	1.80E-03	1.41	9.94E-02	−1.48		***
	*3.769	2.70E-03	−2.03	9.94E-07	−1.40	8.32E-11	−2.12		***		***		***
**Glutamine**	2.476	1.22E-12	−5.44	4.89E-01	<−10	4.15E-01	<−10		***		***		***
	2.47	7.47E-14	−6.42	4.89E-01	<−10	4.15E-01	<−10		***		***		***
	2.458	1.65E-13	−7.26	3.06E-01	<−10	1.52E-14	<−10		***		***		***
	2.45	2.31E-14	−6.11	3.06E-01	<−10	7.15E-17	<−10		***		***		***
	2.44	2.85E-13	−5.77	3.34E-15	<−10	3.58E-16	<−10		***		***		***
	*2.162	5.56E-13	−2.21	1.20E-07	−1.86	4.22E-13	−2.18	4.02E-02	−2.84		***		***
	*2.155	2.34E-12	−3.09	3.35E-02	−4.37	8.06E-13	−5.19		***		***		***
	*2.144	3.80E-14	−3.63	3.35E-02	−4.53	6.05E-15	−7.05		***		***		***
	*2.137	5.28E-11	−2.14	5.88E-12	−2.79	2.00E-13	−4.96		***		***		***
	*2.127	1.62E-08	−1.70	3.35E-02	−1.75	2.51E-12	−3.28		***		***		***
	*3.785		***	3.35E-02	−2.34	1.04E-10	−2.18		***	3.52E-06	−2.99		***
	*3.776	2.70E-03	−2.17	5.61E-13	−4.85	3.51E-08	−5.18	1.80E-03	1.41	9.94E-02	−1.48		***
	*3.769	2.70E-03	−2.03	9.94E-07	−1.40	8.32E-11	−2.12		***		***		***
**Glutathione**	2.972	2.39E-08	3.78		***		***	1.01E-06	−2.48		***		***
	2.553	3.46E-06	3.09		***		***	2.06E-05	−3.01		***		***
	*2.176	1.21E-05	1.74		***		***				***		***
	*2.168	3.50E-06	1.71		***		***	8.25E-04	−1.51		***		***
**Glycophosphocholine**	3.235	2.70E-03	4.35	9.34E-01	4.48	1.65E-01	3.42	6.65E-02	1.92	3.37E-01	−1.19		***
**Isoleucine**	*3.687		***	2.67E-12	4.92		***		***		***		***
	*3.680		***	8.84E-15	2.62		***	7.90E-05	1.84		***		***
	0.944	1.92E-07	−1.63		***	2.70E-03	−2.43		***		***		***
	1.019	1.69E-08	−1.86		***	1.21E-15	−10.39		***		***		***
	1.011	1.80E-08	−1.86		***	1.47E-14	−2.513	1.40E-01	2.77		***	1.18E-01	−6.66
**Lactate**	4.109	2.70E-03	−4.26	4.83E-09	−3.30	2.70E-03	−4.15		***		***		***
**Leucine**	0.975	2.12E-10	−1.79		***	1.09E-13	−2.43		***		***		***
	0.968	2.70E-03	−1.69		***	4.96E-15	−2.36		***		***		***
**Myo inositol** **	4.073	2.37E-02	1.31	2.55E-16	4.00	2.70E-03	3.55	8.06E-09	2.31	3.95E-03	2.88	1.60E-01	−1.48
	3.641	4.49E-05	1.66	1.81E-13	3.76	5.94E-16	3.94	9.83E-09	2.35	4.74E-09	2.32		***
	3.629	2.23E-05	1.79	5.58E-15	3.66	1.08E-15	3.99	8.21E-09	2.36	3.95E-03	2.20		***
	*3.618	7.40E-05	1.71	1.97E-13	5.88	1.15E-15	3.73	2.98E-08	2.30	4.03E-08	2.15	2.93E-05	−1.09
	3.552	3.68E-05	1.67	1.78E-12	1.64	1.04E-15	3.44	6.65E-02	2.54	3.65E-08	2.02	***	***
	3.537	9.32E-05	1.55	9.75E-13	1.43	4.60E-09	3.96		***	1.64E-07	2.37	1.56E-04	−1.11
	3.301	2.70E-03	4.53	1.43E-13	9.69	2.70E-03	4.22	2.44E-09	2.29	7.82E-02	1.79	6.65E-02	−2.73
	*3.29	3.89E-02	1.57	5.47E-02	>10	2.70E-03	2.80	1.25E-08	2.33	3.95E-03	2.38	6.65E-02	−1.59
	*3.278	2.38E-02	1.56	1.00E+00	4.65	3.17E-01	2.45	2.80E-08	2.27	5.47E-02	2.56	4.50E-01	1.46
**Octanoic acid**	1.299		***	8.90E-07	9.69		***		***		***		***
	1.293		***	3.37E-06	>10		***	1.48E-02	1.82		***		***
	1.286		***	3.51E-05	4.65		***		***		***		***
**Phosphocholine**	3.226	2.70E-03	2.62	3.35E-02	−4.52	2.70E-03	2.881	4.02E-02	<−10	3.37E-01	1.10	1.31E-08	>10
**Serine**	3.991	3.42E-09	−2.07	9.50E-15	−5.84	1.37E-13	−3.477	***			***		***
**Succinate**	2.406		***	1.91E-06	−1.91	3.67E-08	2.415	***		5.02E-08	3.53	2.94E-08	4.62
**Threonine**	3.602	8.60E-05	3.62		***		***	1.63E-02	−1.64		***		***
	3.592	4.55E-02	1.56		***		***	4.02E-02	−1.96		***	1.06E-03	>10
	1.341		***	1.76E-01	1.30	3.17E-01	−1.26	2.19E-01	1.26	2.62E-01	−1.42	1.90E-05	−2.03
**UDP species**	5.992	1.57E-07	3.91	***		***		8.04E-07	−3.92		***		***
	5.986	2.64E-06	3.34	***		***		1.27E-05	−3.20		***		***
	5.979	4.76E-08	4.75		***		***	4.15E-06	−2.66	5.65E-07	−3.66		***
	5.969	3.23E-08	4.77		***		***	1.63E-06	−3.06	2.27E-07	−4.38		***
	2.084	6.97E-05	2.47	1.04E-07	<−10	2.70E-03	−3.16	2.22E-05	−4.29	9.80E-06	−6.18		***
**Valine**	1.051	2.70E-03	−1.97	2.88E-07	−1.58	7.50E-18	−4.32		***		***		***
	1.043	2.70E-03	−2.01	3.32E-07	−1.60	4.93E-17	−4.09		***		***		***
	0.992	4.67E-08	−1.95	3.35E-02	−1.54	3.71E-17	−3.92		***		***		***
**Sugar region**	4.387	3.19E-01	9.92		***		***	1.37E-08	−3.21		***		***
	4.381	2.18E-08	3.57		***		***	1.23E-07	−4.17	1.08E-07	−3.94		***
	4.375	2.75E-07	2.29		***		***	2.41E-05	−3.27	8.29E-08	−4.16		***
	4.363	2.03E-07	3.37		***		***	1.44E-06	−3.23		***		***
	4.296	2.01E-12	3.67		***		***	4.25E-10	−2.72		***		***
	4.24	8.62E-11	4.31		***		***	1.28E-08	−1.99		***		***
**UK (m)**	3.173	3.35E-02	<−10	6.51E-17	<−10	4.15E-01	<−10		***		***		***
	3.159	3.74E-16	<−10	8.79E-18	<−10	9.19E-18	<−10		***		***		***
**UK (m)**	2.955	1.58E-14	<−10	4.15E-01	<−10	4.89E-01	****		***		***		***
	2.945		***	4.15E-01	<−10	2.07E-15	−7.40	4.23E-01	<−10	1.38E-08	−5.24		***
	2.936		***	4.15E-01	<−10	7.10E-13	<−10	4.23E-01	<−10	4.02E-02	−8.80		***
**UK (t)**	3.871		***	2.99E-17	−4.89	1.50E-15	−3.08		***		***		***
	3.864	2.70E-03	−3.68	1.62E-15	−7.58	5.42E-15	−3.97		***		***		***
	3.857		***	6.91E-17	−5.73	1.73E-11	−2.39		***		***		***
**UK (m)**	3.826	2.47E-06	3.12		***		***	8.33E-06	−3.27		***		***
	3.803		***		***		***	2.91E-07	−2.96		***		***
	3.798	1.17E-04	3.96		***		***		***		***		***
**UK(s)**	3.772		***		***		***		***			8.47E-05	−1.51
**UK(s)**	3.362		***	3.27E-14	5.77		***	1.06E-04	2.83		***	6.65E-02	−4.72

* 0.01ppm bucket

*** no fold change due to absence of the metabolite in one group

UK = unknown

UK(s) = unknown singlet

UK(t) = unknown triplet

UK(m) = unknown multiplet

**Table 4:** P-values and fold-changes for buckets associated with metabolites in lipophilic cell extracts in the following comparisons: H6C7 and Miapaca-2, H6C7 and Panc-1, H6C7 and AsPC-1, Miapaca-2 and Panc-1, Miapaca-2 and AsPC-1, and Panc-1 and AsPC-1 are listed with the significant buckets highlighted.

Metabolites		Bucket (ppm)	Miapaca-2 *p*-Value	Fold change	Panc-1 *p*-Value	Fold change	AsPC-1 *p*-Value	Fold change	MP *p*-Value	Fold change	MA *p*-Value	Fold change	PA *p*-Value	Fold change
**Phosphatidylglycerol**		3.731	1.80E-12	>10	4.64E-15	>10	1.63E-16	>10		***		***		***

**phosphatidylcholine**		3.358	2.70E-03	<−10	8.23E-02	−6.22	5.83E-07	<−10		***	1.31E-01	1.55		***

**Phosphatidylethanolamine**		3.265	2.70E-03	>10	2.70E-03	>10	2.06E-12	>10	8.55E-04	−1.14	2.35E-01	−1.05	8.19E-02	1.09

**Fatty Acid**	**(−CH=CH-)**	5.307		***	3.01E-06	1.73	7.55E-10	1.72	2.50E-02	1.57	1.31E-01	1.55	7.12E-01	−1.02
		5.276	7.65E-09	1.59	3.89E-05	1.30	1.68E-08	1.43	2.11E-04	−1.22	4.27E-04	−1.12	2.44E-02	1.09
α		2.376	2.13E-08	<−10	2.18E-08	<−10	1.94E-03	<−10	8.55E-04	−1.14		***		***
		2.318	9.16E-07	<−10	1.12E-06	<−10	4.17E-08	−5.57	1.84E-02	1.32	2.28E-01	1.12	6.41E-02	−1.18
		2.282	2.81E-07	<−10	9.69E-07	−2.75	1.11E-06	−2.62	2.01E-02	1.27	2.37E-01	1.10	7.72E-02	−1.16
		2.23	1.52E-02	1.42	3.17E-01	1.03	1.80E-01	1.23	1.17E-05	−1.37	4.68E-04	−1.16	6.98E-04	1.18
		2.198	1.99E-01	1.34	1.99E-01	−1.04	4.45E-01	1.12	7.34E-08	−1.39	1.60E-01	−1.18	6.65E-02	1.18
γ		1.975	1.37E-12	<−10	2.42E-12	−3.56		***		***	1.15E-06	1.25		***
		1.938	9.47E-09	1.60	1.99E-06	1.33	5.50E-02	1.41	6.49E-03	−1.19	4.02E-02	−1.14	8.29E-01	1.04
β		1.801	8.26E-04	3.73	7.21E-08	2.67	7.85E-04	3.27	7.10E-02	−1.65		***		***
		1.569	2.27E-10	<−10	5.30E-03	−1.36	3.35E-02	−1.86	4.19E-01	1.08	1.28E-03	−1.43	9.53E-03	−1.55
**mix**		1.186	5.57E-02	−1.04	7.19E-07	−1.11	3.35E-02	−1.07	3.74E-02	−1.06	5.17E-01	−1.03	1.95E-01	1.03
		1.121		***	1.20E-04	−1.63	4.90E-11	−2.26		***		***	6.68E-04	−1.13

**Cholesterol**	**(C19)**	**0.94	6.23E-07	2.29	3.53E-07	2.58	7.64E-08	2.52	1.32E-01	1.10	2.09E-02	1.14	4.28E-01	1.03
**(C18)**		**0.61	2.15E-07	2.86	2.39E-07	3.01	2.18E-08	2.95	5.40E-01	1.04	1.17E-01	1.09	3.74E-01	1.05

**Aromatic H**		7.008	2.46E-06	4.51	2.70E-03	5.35		***	5.47E-02	1.20	8.03E-01	−1.03	1.31E-01	−1.24
		6.967	1.99E-06	4.62	2.70E-03	5.51			5.47E-02	1.20	7.96E-01	−1.03	3.20E-02	−1.24
		6.936	1.88E-06	4.87	2.92E-08	5.83		***	8.28E-03	1.34		***		***

**FA, cholesterol,** **phospholipid (CH3)**		*0.811	2.14E-05	<−10	3.51E-07	−1.22	2.38E-06	−1.16	3.60E-01	1.03	6.15E-02	1.14	9.48E-03	1.04
		*0.842	2.52E-05	<−10	2.20E-07	−1.17	3.35E-02	−1.10	1.50E-01	−1.47	5.89E-01	−1.07	1.31E-01	1.05
		*0.74	2.04E-05	<−10	1.99E-01	−1.63	1.27E-04	−3.47	1.93E-01	1.14	8.24E-01	1.02		***
		*0.646	2.70E-03	−7.94	1.45E-09	<−10		***		***		***		***

**UK**	**(S)**	7.312	6.23E-07	−4.09	2.11E-06	−3.03	3.35E-02	−3.44		***		***		***
**(S)**		7.066	2.25E-05	−2.13	2.52E-04	−1.71	3.35E-02	−2.07	7.92E-02	1.25		***		***
**(m)**		3.96	2.70E-03	−7.86	5.86E-09	<−10	3.35E-02	−7.22		***		***		***
**(m)**		3.833	3.20E-11	8.14	1.96E-11	8.73	1.51E-15	>10		***		***		***
**(S)**		3.576	2.70E-03	<−10	1.34E-11	<−10	1.44E-11	<−10		***		***		***
**(S)**		3.421	1.52E-08	−6.98	2.88E-08	−5.49	1.90E-08	−5.99		***		***		***
**(m)**		1.506		***		***		***	7.98E-04	−1.13	4.80E-04	−1.15		***
**(m)**		1.447	2.32E-10	2.29	3.25E-11	2.33	5.35E-12	2.02		***		***		***
**(m)**		1.439		***		***	4.57E-11	2.29		***		***		***
**(m)**		1.361	1.48E-07	−2.31	1.81E-05	−1.64	4.18E-07	−1.89	2.65E-03	1.42	3.62E-03	1.27	1.85E-01	−1.10
**(S)**		1.329	1.37E-05	1.96	6.41E-07	2.58		***	2.47E-02	1.95		***		***
**(S)**		0.989	2.70E-03	−2.11	2.70E-03	−1.77	4.05E-08	−1.94		***		***		***

* These peaks contained some overlap. Bucket widths were 0.03 ppm except when indicated by

** which indicates that a 0.005 ppm bucket width was used. Some *p*-values were not reported for buckets experiencing less than 5% variance and these are indicated by

*** UK = unknown, S=singlet, m=multiplet.

**Table 5:** Estimated metabolite concentrations and fold-changes in hydrophilic extracts. Fold-changes were calculated by the treated group concentrations divided by the appropriate control concentrations: H6C7 and Miapaca-2, H6C7 and Panc-1, H6C7 and AsPC-1, Miapaca-2 and Panc-1, Miapaca-2 and AsPC-1, and Panc-1 and AsPC-1.

Metabolites	(ppm)	H6C7	Miapaca-2	Panc-1	AsPC-1
**AXP**	8.61	0.19^a^ (0.056)^b^	0.03 (0.012) −5.60^c^	0.03 (0.013) −5.91	0.04 (0.012)
**Acetamide**	2.024	**	012 (0.060) ***	0.08 (0.020) ***	**
**Acetate**	1.922	0.06 (0.003)	0.17 (0.053) 2.8	0.26 (0.014) 4.36	0.19 (0.108) 3.18
**Alanine**	1.491	0.24 (0.016)	0.10 (0.034) −2.38	0.03 (0.008) −6.97	0.23 (0.068) −1.01
**Aspartate**	2.67(dd)	0.09 (0.008)	0.12 (0.050) 1.25	0.11 (0.023) 1.15	0.43 (0.132) 4.65
**Choline**	3.209 (s)	0.07 (0.082)	0.08 (0.025) −2.6	0.004 (0.0005) −18.3	0.35 (0.121) 5.31
**Creatine**	3.05	0.11 (0.008)	0.01 (0.109) −1.03	0.15 (0.048) 1.35	0.16 (0.053) 1.42
	3.933	0.11 (0.006)	*	0.16 (0.051) 1.49	0.18 (0.063) 1.68
**Glutamate**	2.061 (m)	0.61 (0.029)	*	0.60 (0.172) −1.02	*
	2.163–2.129 (m)	0.56 (0.049)	*	0.59 (0.164) 1.04	*
	2.34–2.37 (m)	0.56 (0.050)	0.47 (0.23) −1.19	0.52 (0.128) 1.08	0.45 (0.149) −1.26
	3.76(m)	*	0.49 (0.267) ***	0.51 (0.136) ***	0.43 (0.14)
**Glutamine**	2.478–2.422 (m)	1.10 (0.050)	0.18 (0.105) −6.13	**	**
**Glutathione**	2.58–2.60(m)	**	0.26 (0.137) ***	**	0.20 (0.047) 1.2
	2.973 (dd)	**	0.27 (0.139) ***	**	*
	3.02 (dd)	**	0.28 (0.158) ***	**	*
**GPC**	3.236	0.03 (0.005)	0.08 (0.021) 1.05	0.17 (0.047) 2.69	0.22 (0.200) 7.89
**Isoleucine**	0.944 (t)	0.12 (0.007)	0.07 (0.031) −1.91	0.07 (0.022) −1.68	0.07 (0.022) −1.72
	1.011 (d)	0.12 (0.006)	0.05 (0.038) −2.19	0.07 (0.024) −1.71	0.07 (0.02) −1.82
**Lactate**	1.342 (d)	0.81 (0.080)	0.13 (0.009) −6.04	0.20 (0.036) −4.1	0.21 (0.036) −3.79
	4.1 (q)	0.79 (0.079)	0.13 (0.006) −6.16	0.20 (0.036) −4.02	*
**Leucine**	0.945(d)	0.13 (0.004)	0.06 (0.028) −2.08	0.07 (0.022) −1.82	0.07 (0.024) −1.77
	0.975(d)	0.13 (0.005)	0.06 (0.027) −2.1	0.08 (0.021) −1.9	0.07 (0.023) −1.83
**Myo-inositol**	4.05 (t)	0.31 (0.015)	0.41 (0.138) 1.32	1.04 (0.250) 3.32	1.70 (0.567) 2.06
	3.6389–3.616 (t)	0.29 (0.023)	0.37 (0.116) 1.27	0.98 (0.241) 3.34	1.55 (0.474) 5.28
	3.551–3.549 (dd)	0.35 (0.028)	0.44 (0.143) 1.25	1.13 (0.289) 3.2	1.78 (0.560) 5.02
	3.287–3.276 (t)	0.35 (0.017)	0.43 (0.139) 1.23	1.13 (0.292) 3.27	1.70 (0.516) 4.92
**Octanoic acid**	0.85(t)	**	0.03 (0.005) ***	0.05 (0.002) ***	**
**PC**	3.226	0.07 (0.006)	0.15 (0.084) 2.03	0.01 (0.002) −7.15	0.26 (0.088) 3.53
**Phospho creatine**	3.048	0.08 (0.004)	0.03 (0.010) −2.63	0.13 (0.025) 1.64	0.08 (0.026) 1.04
**Serine**	3.992 (dd)	0.24 (0.026)	**	**	**
	3.94(dd)	0.24 (0.021)	**	**	**
	3.84 (dd)	0.22 (0.019)	**	**	**
**3-hydroxy-isovalerate**	1.255 (s)	0.07 (0.002)	**	**	**
**Succinate**	2.4 (s)	0.02 (0.002)	0.005 (0.002) −4.65	**	0.09 (0.035) 4.02
**Threonie**	1.32(d)	0.13 (0.005)	0.13 (0.062) 1	0.10 (0.034) −1.33	0.09 (0.041) −1.43
	3.592 (d)	0.11 (0.008)	*	*	*
**Valine**	0.993 (d)	0.12 (0.005)	0.05 (0.025) −2.3	0.06 (0.019) −1.93	0.04 (0.012) −2.85
	1.043 (d)	0.12 (0.005)	0.05 (0.023) −2.33	0.06 (0.019) −1.94	0.04 (0.012) −2.96
	3.60 (d)	0.12 (0.009)	*	*	*

* overlap

** not detected

*** fold change not reported due to the absence of the metabolite in one group

a concentration of metabolite (mM)

b standard deviation of concentration (mM) in parentheses

c fold-change. A negative value indicates that the treatment group concentration was lower than in the control group concentration. In this case, the treatment group concentration was divided by the control group concentration and then the negative of the inverse of this ratio was reported in the table.

## Abbreviations

BMRB: Biological Magnetic Resonance Data Bank
FBS: Fetal Bovine Serum
GalNAc: N-Acetylgalactosamine
GC-MS: Gas Chromatography Mass Spectrometry
GlcNAc: N-Acetylglucosamine
H6C7: Immortalized Human Pancreatic Ductal Epithelial Cell Line
HPDE6-E6E76c7: HDPE Human Ductal Pancreatic Epithelial
LC-MS: Liquid Chromatography Mass Spectrometry
NMR: Nuclear Magnetic Resonance
NAA: N-Acetylaspartic Acid
NOESY: Nuclear Overhauser Effect Spectroscopy
PC: Principal Component
PCA: Principal Component Analysis
TCA cycle: Tricarboxylic Acid Cycle
TSP: Trimethylsilylpropionate
UDP: Uridine Diphosphate
