# Supplementary material for: Metabolic Profiling Comparison of Human Pancreatic Ductal Epithelial Cells and Three Pancreatic Cancer Cell Lines using NMR Based Metabonomics
Source: J Mol Biomark Diagn. Author manuscript; Available in PMC 2015 Nov 23. (PMC4655885; doi:10.4172/2155-9929.S3-002)
Supplement: supplemental [file NIHMS711669-supplement-supplemental.pdf]

Supplementary Material for:

Metabolic profiling comparison of human pancreatic ductal epithelial cells and three pancreatic cancer cell lines using NMR based metabonomics.

Miki Watanabe[a], Sulaiman Sheriff[b], Kenneth B. Lewis[a], Junho Cho[a], Stuart L. Tinch[a]
Ambikaipakan Balasubramaniam[b,c,d] and Michael A. Kennedy[a*]

Contents:

Supplemental Figure 1. (A) Two-dimensional PC1 versus PC2 loadings plot corresponding to the scores plot of hydrophilic cell extract data in Fig 3A. (B) Two-dimensional PC1 versus PC2 loadings plot corresponding to the scores plot of lipophilic cell extract data in Fig 3B.

Supplemental Figure 2. Two-dimensional PC1 versus PC2 scores plot and the loadings plot comparing hydrophilic extracts of A)H6C7 and Miapaca-2, B)H6C7 and Panc-1, C)H6C7 and AsPC-1, D)Miapaca-2 and Panc-1, E)Miapaca-2 and AsPC-1, F)Panc-1 and AsPC-1.

Supplemental Figure 3. Two-dimensional PC1 versus PC2 scores plot and the loadings plot comparing lipophilic extracts of A)H6C7 and Miapaca-2, B)H6C7 and Panc-1, C)H6C7 and AsPC-1, D)Miapaca-2 and Panc-1, E)Miapaca-2 and AsPC-1, F)Panc-1 and AsPC-1.

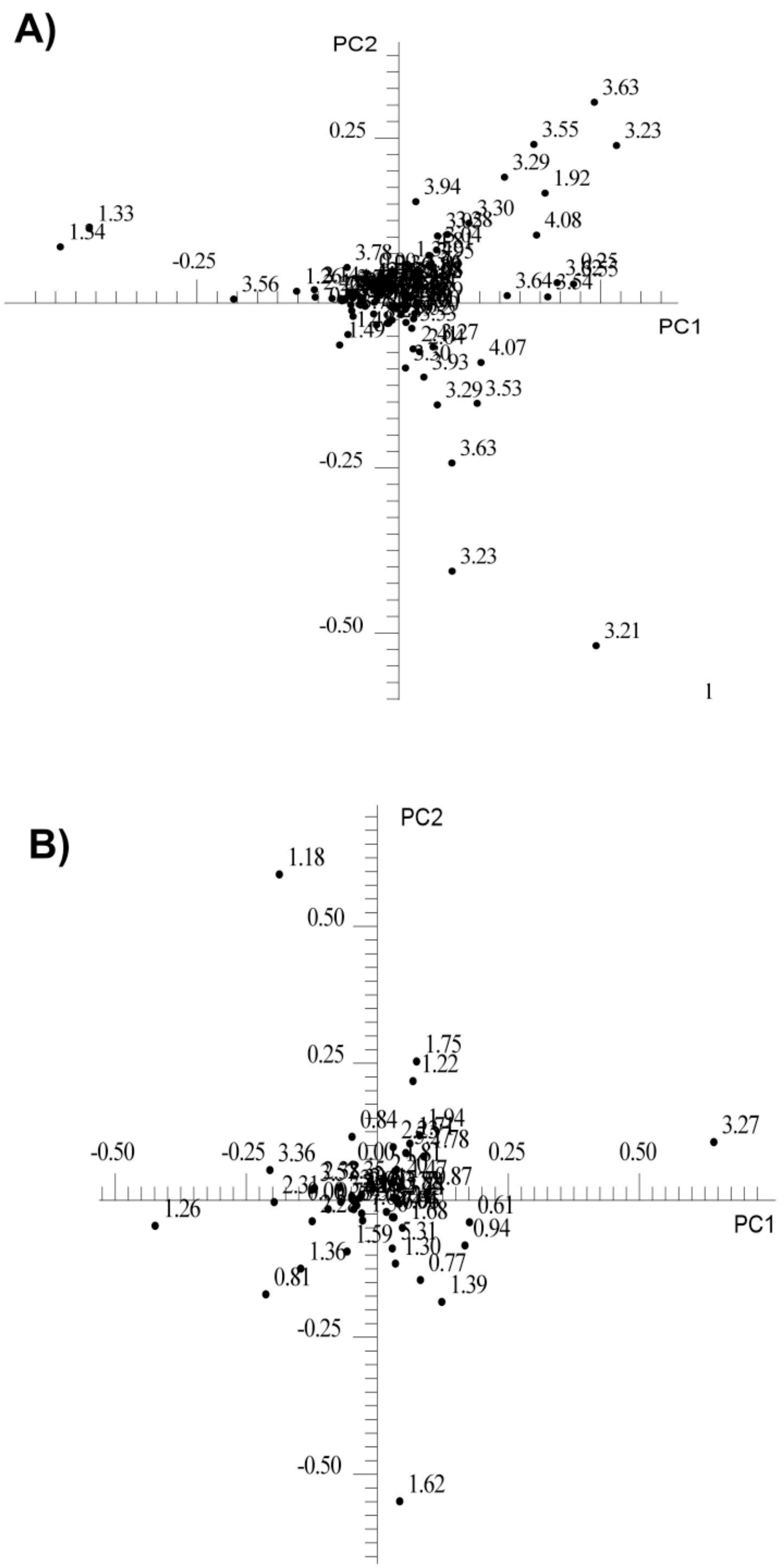

Supplemental Figure 1. (A) Two-dimensional PC1 versus PC2 loadings plot corresponding to the scores plot of hydrophilic cell extract data in Fig 3A. (B) Two-dimensional PC1 versus PC2 loadings plot corresponding to the scores plot of lipophilic cell extract data in Fig 3B.

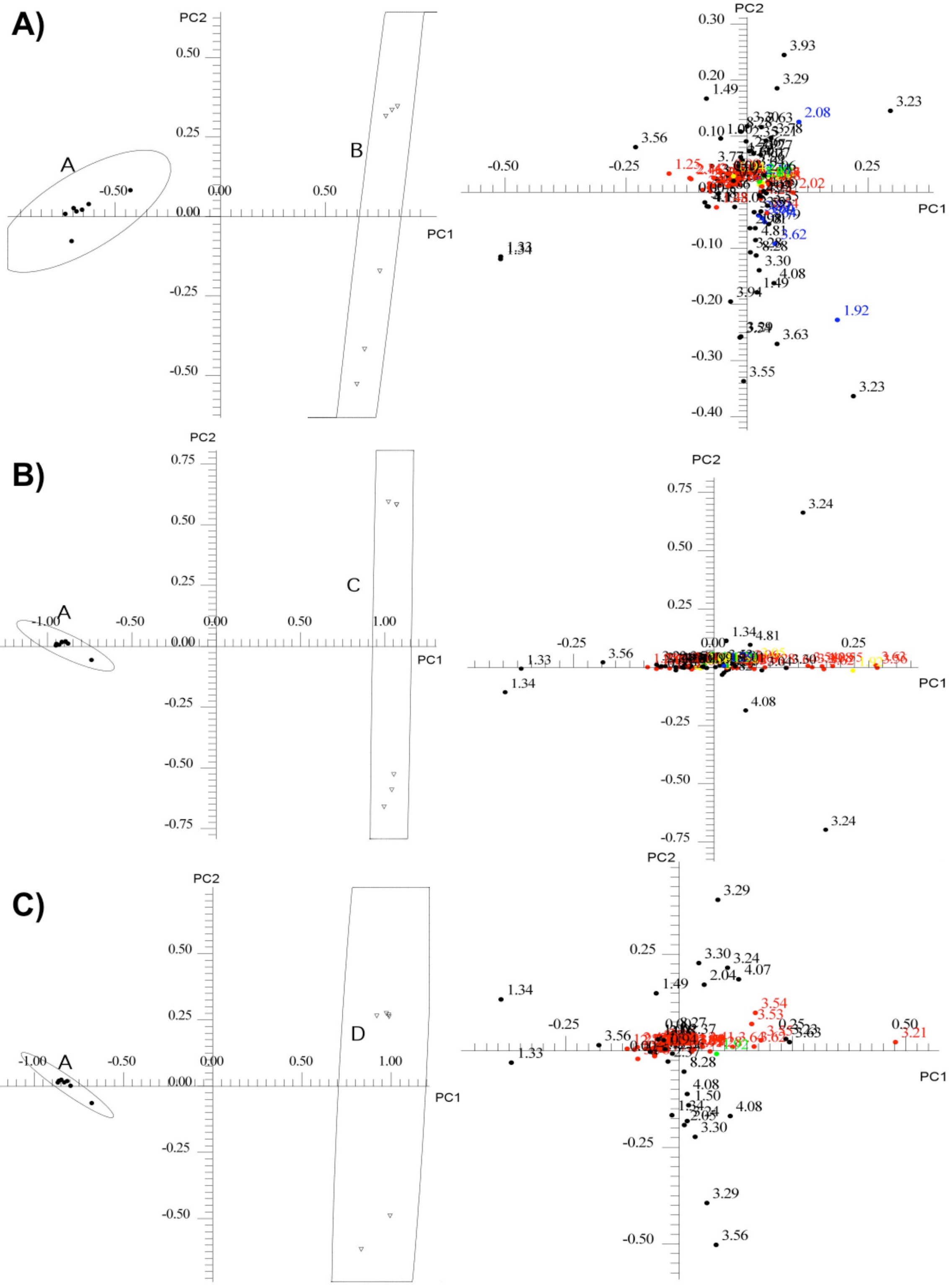
A)
B)
C)
PC1
PC2
A
B
C
D

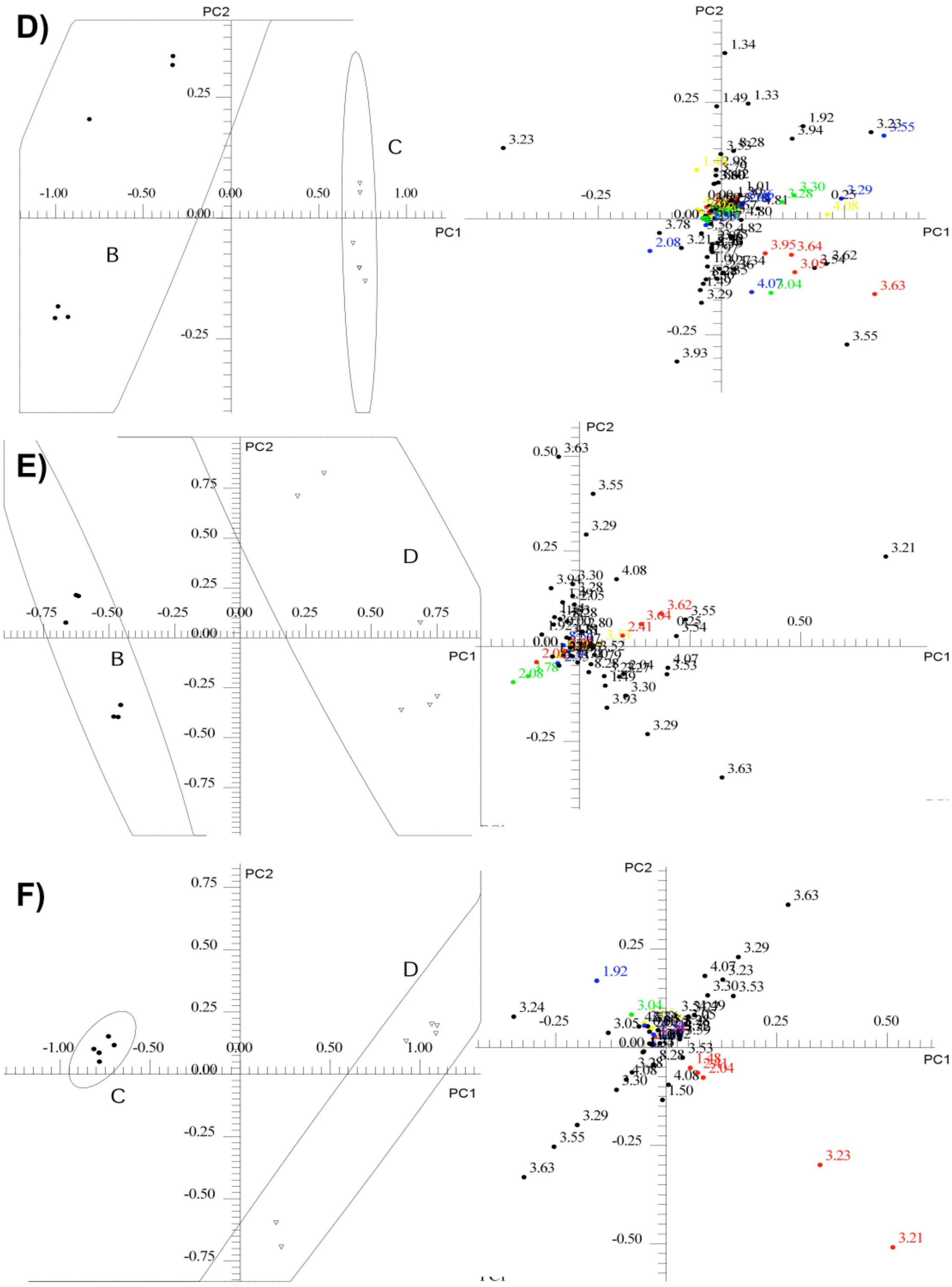

Supplemental Figure 2. Two-dimensional PC1 versus PC2 scores plot and the loadings plot comparing hydrophilic extracts of A)H6C7 and Miapaca-2, B)H6C7 and Panc-1, C)H6C7 and AsPC-1, D)Miapaca-2 and Panc-1, E)Miapaca-2 and AsPC-1, F)Panc-1 and AsPC-1. In the scores plot, the 95% confidence interval for the two clusters of data points were indicated with oval lines. The Bonferroni-corrected *p*-values from 0.005 ppm buckets for hydrophilic cell extracts were determined for each comparison and listed in Table 2 The loading plots are color-coded according to bucket *p*-values: Black (>α-value), Blue (α-value - 10^{-5}), Green (10^{-5}- 10^{-6}), Yellow (10^{-6} - 10^{-7}), Red (10^{-7}- 0).

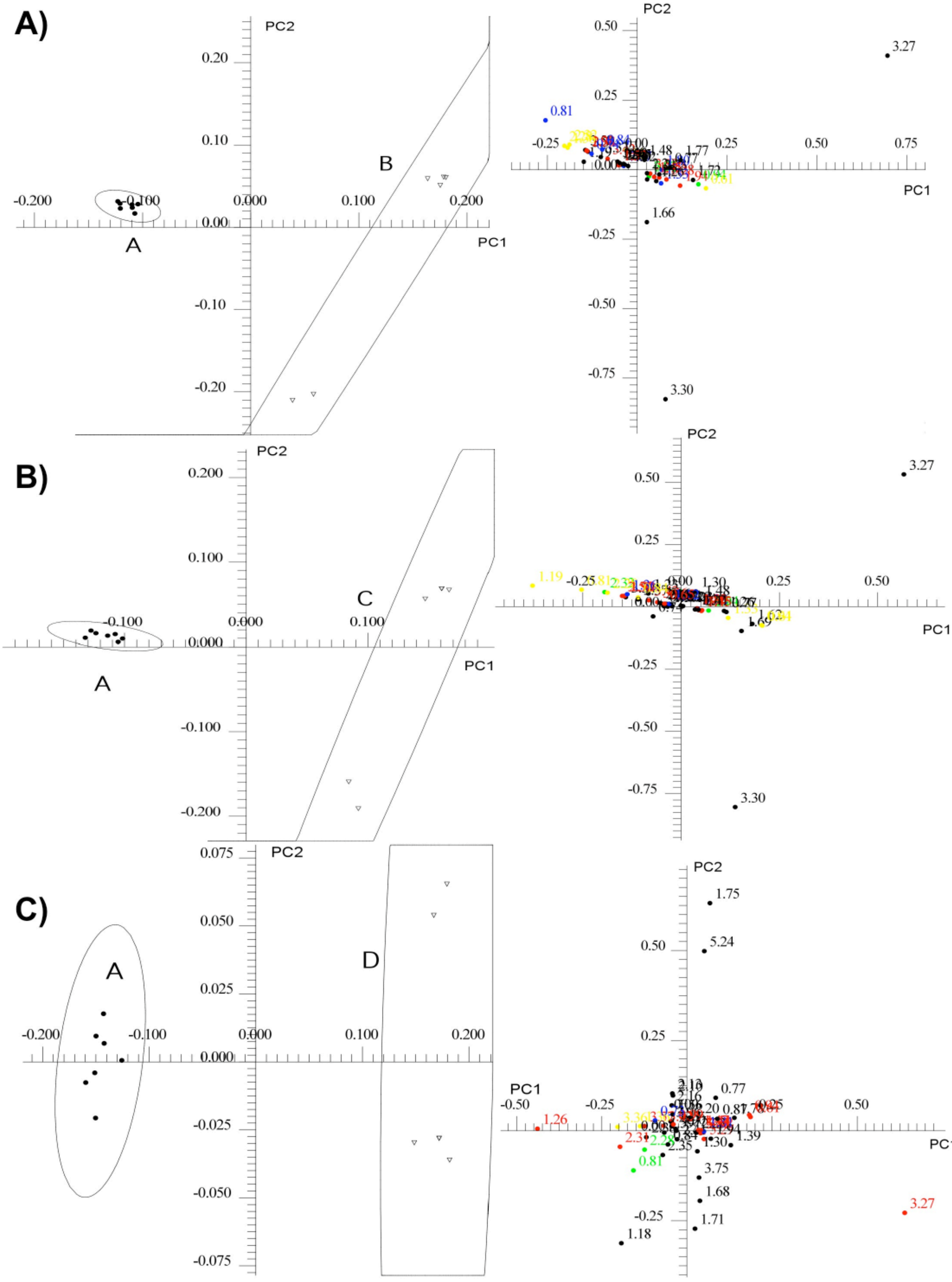

A)
PC2
PC1
0.20
0.10
0.00
-0.10
-0.20
-0.200
-0.100
0.000
0.100
0.200
A
B
0.50
0.25
-0.25
-0.50
-0.75
0.75
3.27
0.81
1.48
1.77
1.73
1.66
3.30
B)
0.200
0.100
0.000
-0.100
-0.200
C
1.19
1.30
1.62
C)
0.075
0.050
0.025
-0.025
-0.050
-0.075
D
1.75
5.24
0.77
1.26
0.81
1.39
1.30
2.35
3.75
1.68
1.71
1.18

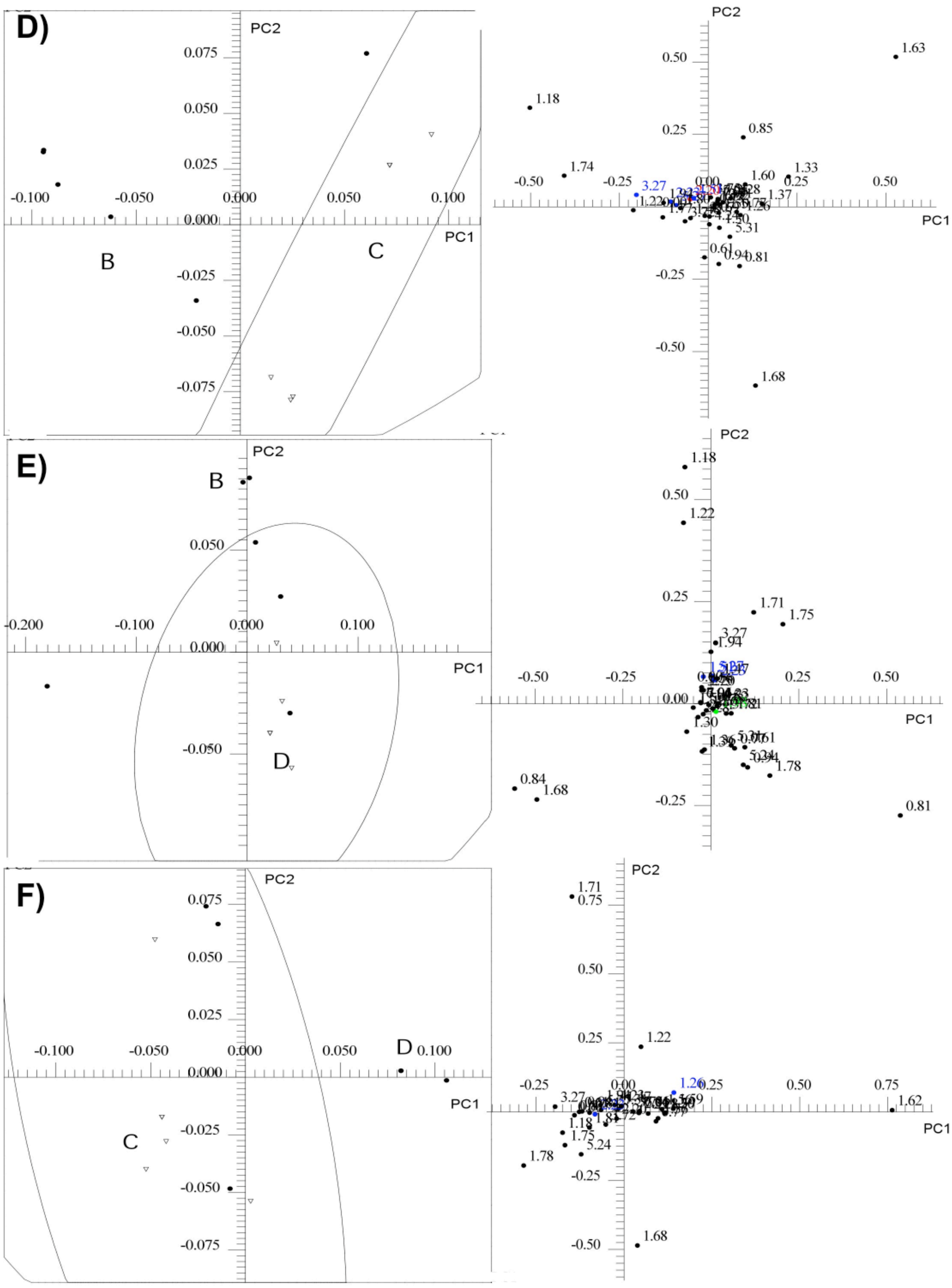

Supplemental Figure 3. Two-dimensional PC1 versus PC2 scores plot and the loadings plot comparing lipophilic extracts of A)H6C7 and Miapaca-2, B)H6C7 and Panc-1, C)H6C7 and AsPC-1, D)Miapaca-2 and Panc-1, E)Miapaca-2 and AsPC-1, F)Panc-1 and AsPC-1. In the scores plot, the 95% confidence interval for the two clusters of data points were indicated with oval lines. The Bonferroni-corrected *p*-values from 0.03 ppm buckets for lipophilic cell extracts were determined for each comparison and listed in Table 2. The loading plots are color-coded according to bucket *p*-values: Black (>α-value), Blue (α-value - 10^{-5}), Green (10^{-5}- 10^{-6}), Yellow (10^{-6} - 10^{-7}), Red (10^{-7}- 0).
